# Network meta-analysis of pharmacological treatments for idiopathic pulmonary fibrosis: evaluating effects on lung function

**DOI:** 10.3389/fphar.2026.1761899

**Published:** 2026-02-23

**Authors:** Yajie Yin, Xinhui Wu, Zhihao Liu, Yinru Luo, Mi Jing, Kefeng Jing, Qiuyuan Li, Fei Wang, Ju Huang

**Affiliations:** 1 Hospital of Chengdu University of Traditional Chinese Medicine, Chengdu, China; 2 Department of Respiratory Medicine, Hospital of Chengdu University of Traditional Chinese Medicine, Chengdu, China

**Keywords:** idiopathic pulmonary fibrosis, network meta-analysis, pharmacological treatments, pulmonary function, therapeutic efficacy

## Abstract

**Background:**

Idiopathic pulmonary fibrosis (IPF) is a chronic, progressive fibrotic interstitial lung disease of unknown cause. Its main feature is a steady decline in lung function, which is also the primary target for treatment. Existing research has investigated various drugs to slow IPF progression, but their effectiveness and how they affect key pulmonary function indicators need to be systematically evaluated and analysed.

**Methods:**

This systematic review and network meta-analysis searched eight databases to identify randomised controlled trials assessing the effects of various pharmacological treatments on lung function in patients with IPF. The risk of bias in the included studies was evaluated using tools from the Cochrane Handbook. Network meta-analysis was conducted using Stata 19.0 and R 4.5.1 software. The study protocol was registered in PROSPERO (CRD420251148658).

**Results:**

This study included 121 publications comprising 162 studies, covering 16,525 IPF patients across nine countries. The overall risk of bias assessment showed that while most studies had a low risk of bias in random sequence generation, concerns regarding allocation concealment and blinding were identified in a substantial proportion of the included studies. Network meta-analysis revealed that Nerandomilast was the most effective intervention for improving Forced Vital Capacity (FVC) (SUCRA: 98.85%). N-acetylcysteine (NAC) combined with Roxithromycin (RXM) was the most effective intervention for improving Vital Capacity (VC) (SUCRA: 88.8%) and Forced Expiratory Volume in 1 s/Forced Vital Capacity (FEV1/FVC) (SUCRA: 97.45%). Ambroxol was the most effective intervention for improving Total Lung Capacity (TLC) (SUCRA: 82.52%), while Thalidomide was the most effective intervention for improving Diffusing Capacity of the Lung for Carbon Monoxide (DLCO) (SUCRA: 90.93%).

**Conclusion:**

The results suggest that drugs targeting different pulmonary function parameters have corresponding mechanisms of action. Nerandomilast shows potential for improving FVC, while NAC combined with RXM may enhance VC and FEV1/FVC. Ambroxol appears effective in increasing TLC, and Thalidomide may boost DLCO. Nonetheless, these findings need validation through higher-quality studies in the future. Additionally, future research should examine the long-term effectiveness of new drugs like Nerandomilast and Pamrevlumab, while also improving comprehensive assessments of synergistic changes across various pulmonary function indicators.

**Systematic Review Registration:**

https://www.crd.york.ac.uk/PROSPERO/view/CRD420251148658.

## Background

Idiopathic pulmonary fibrosis (IPF) is a chronic, progressive fibrotic interstitial lung disease of unknown cause (Raghu et al., 2022). Its prevalence and incidence increase with age, mainly affecting middle-aged and older adults (Mortimer et al., 2020), with a higher occurrence in males (Podolanczuk et al., 2023). The median survival after diagnosis is 2–4 years (Ballester et al., 2019). The primary pathological process of IPF starts from repeated injury and impaired repair of alveolar epithelial cells influenced by multiple risk factors, including smoking, dust, and genetic susceptibility (Hasegawa et al., 2024). This process leads to cell death and the release of pro-inflammatory and pro-fibrotic factors, causing chronic inflammation and immune dysregulation (Crosby and Waters, 2010; Bringardner et al., 2008). Persistent inflammatory signals activate and transform pulmonary interstitial fibroblasts into myofibroblasts (King et al., 2011). These cells produce excessive extracellular matrix (ECM) components like collagen (Moss et al., 2022), leading to abnormal ECM buildup and remodelling, which ultimately forms scar tissue (Geng et al., 2022; Deng et al., 2020). After irreversible structural damage to lung tissue, abnormalities in blood vessel growth and impaired gas exchange develop. Damage to microvascular endothelial cells worsens local inflammation and blood clotting issues (Lu et al., 2025; Farkas et al., 2011). At the same time, tissue hypoxia, caused by gas exchange problems, further stimulates fibroblast activation and ECM production, creating a vicious cycle (Yang et al., 2023; Liu et al., 2022). This ongoing destruction of lung structure and fibrosis ultimately results in typical pulmonary dysfunction: decreased lung compliance and limited expansion due to the replacement of functional lung tissue with scar tissue, appearing as reduced lung volumes (Richeldi et al., 2012; Plantier et al., 2018). At the same time, thickening of the alveolar-capillary barrier and destruction of capillaries severely compromise gas exchange, significantly reducing carbon monoxide diffusion capacity (van Noord et al., 1989; Ley et al., 2012). However, the pathology mainly restricts lung expansion rather than blocking airways, resulting in a smaller decline in expiratory flow rates compared to the reduction in forced vital capacity (FVC) (Plantier et al., 2018), characteristic of a restrictive ventilatory pattern.

In response to the complex pathogenesis of IPF, existing research has explored multiple pharmacological treatment strategies (Gharaee-Kermani et al., 2007). Pirfenidone (PFD) and nintedanib are currently the main drugs approved for IPF treatment (Spagnolo et al., 2021), delaying pulmonary fibrosis progression by inhibiting the inflammation-fibrosis network (Tang et al., 2022) and blocking fibroblast proliferation (Tu et al., 2024), respectively. Beyond these, the antioxidant N-acetylcystine (NAC), as a glutathione precursor, not only directly scavenges reactive oxygen species (ROS) to reduce oxidative stress damage (Qi Z. et al., 2024) but also suppresses the NF-κB pathway, decreasing the release of pro-inflammatory factors like IL-6 and IL-8, thereby providing anti-inflammatory and cytoprotective effects (Raghu et al., 2021; Santus et al., 2024). Macrolide antibiotics can inhibit the release of inflammatory mediators, modulate the inflammatory microenvironment, and indirectly slow the fibrotic process (Bosnar et al., 2009). Simtuzumab, a targeted therapy for LOXL2, directly targets established old scar tissue to promote remodelling of lung tissue structure (Espindola et al., 2023).

Although the drugs listed above target the complex pathophysiology of IPF through various mechanisms, providing patients with multiple treatment options, and although network meta-analyses have attempted to compare their effectiveness in slowing lung function decline, these analyses did not include all drugs and focused only on a single indicator—FVC (Canestaro et al., 2016; Rochwerg et al., 2016; Pitre et al., 2022). However, lung function impairment in IPF is multidimensional, involving reductions in lung volume, impaired gas exchange, and changes in ventilation efficiency. To offer more comprehensive evidence for clinical practice and address the limitations of existing network meta-analyses, we conducted the study “Network Meta-Analysis of Pharmacological Treatments for Idiopathic Pulmonary Fibrosis: Evaluating Effects on Lung Function.” This study aims to systematically assess and compare the effects of PFD, nintedanib, and other IPF treatments with different mechanisms on pulmonary function indicators.

## Methods

This study was reported following the Preferred Reporting Items for Systematic Reviews and Meta-Analyses (PRISMA) extension statement for network meta-analysis (NMA) (Hutton et al., 2015). The PRISMA NMA checklist is seen in Supplementary Table S1: PRISMA 2020 checklist. The protocol was registered in PROSPERO (Registration Number: CRD420251148658).

### Information source and search strategy

Two investigators independently conducted comprehensive literature searches following a predefined strategy. The search period extended from the start of each database until 5 June 2025. The following eight databases were searched: PubMed, Embase, Web of Science, Cochrane Library, China National Knowledge Infrastructure (CNKI), Wanfang, VIP, and China Biomedical Literature Service System (SinoMed). The search strategy focused on “IPF,” “Drug therapy,” and “specific drug names,” utilising both subject headings and free-text terms. Key search terms included: “Idiopathic Pulmonary Fibrosis, Pulmonary Fibroses, Idiopathic, Drug therapy, Pharmacotherapy, pirfenidone, nintedanib, N-Acetylcysteine, sildenafil, colchicine, bosentan, simtuzumab, and others.” Different search strategies were customised according to each database’s characteristics. Additionally, references cited in the included studies were traced to identify further relevant literature. The search was not restricted by language. Our search strategy is presented in Supplementary Table S2.

#### Inclusion and exclusion criteria

This study’s inclusion and exclusion criteria follow the Participant, Intervention, Comparison, Outcome, and Study Type (PICOS) framework. Inclusion and exclusion criteria are presented in Table 1.

**TABLE 1:** Inclusion and exclusion criteria.

​	Inclusion criteria	Exclusion criteria
Patients (P)	Adult patients diagnosed with IPF; Diagnostic criteria must comply with international guidelines for IPF diagnosis. (Raghu et al., 2011)	Patients with other types of interstitial lung disease (ILD) not classified as IPF, such as connective tissue disease-associated ILD, allergic pneumonitis, and pneumoconiosis; Children or adolescents with IPF; Patients with other severe underlying lung diseases, such as COPD, asthma, or lung cancer, may significantly affect lung function assessment.
Intervention (I)	Accept any evaluated drug treatment regimen for IPF; The dosage, treatment duration, and route of administration comply with the study protocol or standard clinical practice.	No medication or treatment has been administered, such as pulmonary rehabilitation, oxygen therapy, or lung transplantation.
Comparison (C)	Direct or indirect comparisons between different IPF drug treatment regimens, including: drug vs. drug, drug vs. placebo, combination therapy vs. monotherapy or placebo.	No control group design
Outcome (O)	Pulmonary function indicators: At least one of the following quantitative data points must be reported. 1. Forced Vital Capacity (FVC) 2. Vital Capacity (VC) 3. Total Lung Capacity (TLC) 4. Diffusing Capacity of the Lung for Carbon Monoxide (DLCO) 5. Forced Expiratory Volume in 1 s/Forced Vital Capacity (FEV1/FVC)	Studies that did not report any pulmonary function parameters. Studies that reported only qualitative descriptions or non-quantitative data for pulmonary function parameters. Studies that reported only other outcome measures, such as survival rates, quality of life questionnaires, or imaging scores.
Study Design (S)	Randomised controlled trials, whether blinded or unblinded, multicenter, and pre-registered.	Non-randomised studies, such as observational cohort studies, case-control studies, and case reports; Non-controlled studies; Reviews, meta-analyses, editorials, conference abstracts, etc.,; Studies with unavailable full text or extractable data; Duplicate publications.

### Study selection

After completing the literature search, all retrieved bibliographic records were imported into EndNote 2025 software for initial management and removal of duplicates. Then, two investigators independently screened the records based on predefined inclusion and exclusion criteria (Table 1). The screening process had two stages: First, investigators independently examined the titles and abstracts of all records, excluding studies that clearly did not meet the criteria. Second, they retrieved and reviewed the full texts of records considered potentially eligible or uncertain from the first stage, ultimately deciding to include randomised controlled trials. Throughout the process, both investigators independently recorded their screening decisions. Any disagreements were resolved through discussion; if necessary, a third researcher helped make the final decision.

### Extraction and analysis

Two investigators independently extracted detailed data from the included studies using Excel 2019. This included basic study information (first author’s name, publication year, first author’s country of affiliation), trial registration number, study center type (single-center or multi-center), patient characteristics (gender, age, sample size), treatment details (intervention, dosage, route of administration, timing), outcome measures, and the number of adverse events. Lung function data were consistently recorded using means and standard deviations (SD). When only the standard error of the mean (SEM) was available, raw data were converted to SD based on statistical principles. For studies where outcome data were presented exclusively in graphical form, data points were extracted independently by two investigators using Origin 2021 software. The two extracted datasets were then cross-verified. Any discrepancy exceeding a pre-specified tolerance of 0.005 units was resolved by re-extraction and consensus. If key data were missing or inadequately described in a publication, the first author of this study attempted to contact the original or corresponding author via email to request the data. The study was excluded if the required data could not be obtained after these attempts. After completing independent data extraction, two investigators cross-checked all entries. Any disagreements were resolved by reviewing and discussing the original publication. If disputes persisted, a third researcher assisted with adjudication.

### Risk of bias assessment

Two investigators independently evaluated study quality using the Cochrane Risk of Bias tool (Higgins et al., 2011) in Review Manager 5.4 software, assessing seven criteria: Selection bias (random sequence generation, allocation concealment), performance bias (blinding of participants and personnel), detection bias (blinding of outcome assessment), incomplete outcome data, reporting bias (selective reporting), and other biases. Each item was rated as low risk (method properly applied), unclear risk (method unclear), or high risk (method improperly applied or not applied). This evaluation was performed independently by two investigators and cross-checked. Disagreements were resolved through discussion between the two; if no consensus was reached, a third researcher helped make the final decision.

### Data synthesis and analysis

Data meeting inclusion criteria were analysed using Stata 19.0 and R 4.5.1. First, a network relationship diagram was created in Stata 19.0 to visualise the comparison network among interventions. Nodes represented interventions, while lines indicated direct comparisons between two interventions. Thicker lines signified a higher number of studies conducting direct comparisons. Funnel plots were generated to evaluate publication bias through visual symmetry assessment. To enhance clinical interpretability, standardized mean differences (SMDs) were converted to mean differences (MDs) using Stata 19.0. This conversion was necessary only for outcomes reported in inconsistent units across studies, such as Forced Vital Capacity (FVC) being reported in both liters and percent predicted, which prevents direct MD pooling. For outcomes consistently reported in the same unit, MDs were calculated directly. The conversion relied on a common standard deviation (SD) as the scaling factor. We used the pooled baseline SD from all placebo groups within each outcome network for this purpose, assuming comparable measurement variability across treatment arms. The metancommand in Stata with the eformoption was applied to obtain MDs with 95% credible intervals, presenting results in clinically meaningful units. Analysis was performed in R 4.5.1 using the gemtc, readxl, and ggplot2 packages: a random-effects model was built, utilising a Markov Chain Monte Carlo (MCMC) algorithm with 50,000 iterations, including 20,000 warm-up iterations to optimise the initial values. Multi-arm trials were accounted for by adjusting the within-study covariance structure to model the correlation between treatment comparisons with a common comparator. Convergence of the MCMC chains was assessed using the Gelman-Rubin-Brooks statistic, with a potential scale reduction factor (PSRF) of less than 1.05 indicating successful convergence. Additionally, visual inspection of trace plots was performed to ensure adequate mixing and stationarity of the chains. These diagnostic tests confirmed that convergence was achieved for all model parameters. Posterior statistics were extracted post-analysis to obtain effect sizes with 95% credible intervals. These results created a forest plot to illustrate the comparative effects between interventions and controls. For closed-loop evidence, node splitting identified local inconsistencies and measured the divergence between direct and indirect evidence, with *P* < 0.05 as the significance threshold. A league table was also developed to systematically display effect sizes and credible intervals for all pairwise intervention comparisons. Efficacy ranking was based on the Sum of Ranked Probabilities Curve Area (SUCRA): the SUCRA value (ranging from 0% to 100%, with higher values indicating better efficacy), and a SUCRA ranking plot was generated to compare the interventions’ relative strengths visually. To evaluate the effects of baseline age and follow-up duration as potential effect modifiers on pulmonary function outcomes and to test the stability of the results, a network meta-regression model was further applied for analysis.

## Results

### Literature screening process and results

**FIGURE 1:**
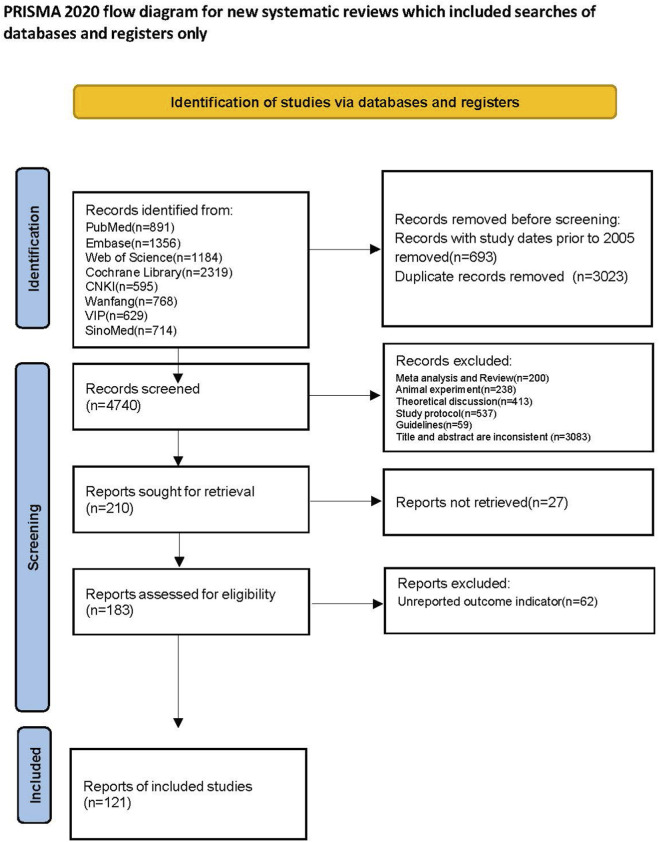
Flow diagram of the study-search process.

Based on the search strategy, we initially retrieved 8,456 articles from eight databases (PubMed: 891, Embase: 1,356, Web of Science: 1,184, Cochrane Library: 2,319, CNKI: 595, Wanfang: 768, VIP: 629, SinoMed: 714). To focus on the critical period of modern diagnostic criteria and core drug therapy development for IPF, we first excluded 693 articles published before 1 January 2025, and then removed 3,023 duplicate records. The remaining 4,740 articles advanced to the following screening stage. Next, we excluded 1,447 articles based on study type: 200 meta-analyses and reviews, 238 animal studies, 413 theoretical discussions, 537 research protocols, and 59 guidelines. We then screened the titles and abstracts of the remaining 3,293 articles. Using specific inclusion and exclusion criteria, we eliminated 3,083 articles, leaving 210 for full-text review. During full-text assessment, we excluded 27 articles because full texts were unavailable. After a thorough review, we excluded 62 articles that did not report relevant outcome measures. Ultimately, 121 articles met all inclusion criteria (Demedts et al., 2005; Zhang et al., 2005; Bai and Wu, 2006; Liu and Qiu, 2006; Wang and Wang, 2006; Wang and Li, 2006; Nan, 2007; Zhigang et al., 2008; Jiang et al., 2009; Zhu et al., 2009; Huang et al., 2010; Taniguchi et al., 2010; Zuo et al., 2010; Long et al., 2011; Richeldi et al., 2011; Yang et al., 2011; Zhu and Chen, 2011; Ge et al., 2012; Homma et al., 2012; Yang et al., 2012; Ying-kun et al., 2012; Yu, 2012; Zhang, 2012; Lu and Gao, 2013; Shi et al., 2013; Li et al., 2014; Li, 2014; Martinez et al., 2014; Richeldi et al., 2014; Zhang, 2014; Chuan-hai et al., 2025; Fu et al., 2015; Huang et al., 2015; Huang, 2015; Jiang, 2015; Jin, 2015; Li et al., 2015; Liu, 2015; Shen et al., 2015; Xian-jun et al., 2015; Zhao, 2015; Behr et al., 2016; Chuanhai and Chenghong, 2016; Li et al., 2016; Liu et al., 2016; Liu, 2016; Lu and Fa, 2016; Tian, 2016; Xu et al., 2016; Azuma et al., 2017; Chen and Feng, 2017; Guo, 2017; Mu et al., 2017; Raghu et al., 2017a; Wang, 2017; Xu, 2017; Chai, 2018; Kai-chun et al., 2018; Kolb et al., 2018; Li H., 2018; Li J., 2018; Mao, 2018; Meng, 2018; R et al., 2018; Shi et al., 2018; Vancheri et al., 2018; Cao et al., 2018; Chen and Guo, 2019; Chen and Guo, 2019; Guo and Wang, 2019; Jianying et al., 2019; Liang et al., 2019; Wang et al., 2019; Wu and Pan, 2019; Yang and Ren, 2019; Zhao and Xu, 2019; Zhu and Zhu, 2019; Lancaster et al., 2020; Liang, 2020; Lin, 2020; Richeldi et al., 2020a; Richeldi et al., 2020b; Song et al., 2020; Zou and Yin, 2020; Zhang and Chen, 2020; Zhang and Liu, 2020; Zhao, 2020; Su-e W, 2021; Tao, 2021; Wang et al., 2021; Zhou et al., 2021; Chen and Yin, 2022; Deng, 2022; Guo, 2022; He, 2022; Song et al., 2022; Xia and Lv, 2022; Yang, 2022; Chanchan, 2023; Chen, 2023; Hu, 2023; Wang et al., 2023; Wang, 2023; Zhao and Gao, 2023; Chen et al., 2024; Chen, 2024; Huanqin and Wang, 2024; Kong, 2024; Lei and Liu, 2024; Qi et al., 2024b; Raghu et al., 2024; Wang, 2024; Wu, 2024; Yue, 2024; Zhang, 2024; Zhao and Luo, 2024; Zhu, 2024; Liu et al., 2025; Zhou et al., 2024; Zulipeiya and Maimaiti, 2024; Maher et al., 2025; Zhang et al., 2025) (Figure 1).

### Description of included studies

The final 121 studies included in this research were independently analysed when a single publication involved multiple trials or intervention time points, resulting in a total of 162 studies. Among these, 25 studies (18.1%) reported clinical trial registration numbers, and 28 studies (20.3%) used a multicenter design. The studies took place in nine countries, enrolling 16,525 IPF patients: 8,719 in intervention groups and 7,806 in control groups. Participants were mostly male (intervention: 5,951 males, 2,736 females; control: 5,253 males, 2,512 females), with baseline ages ranging from 35 to 75 years across both groups. A total of 162 studies assessed 24 interventions (including placebo). The most common interventions studied were NAC (52 studies), PFD (27 studies), and nintedanib (24 studies). Follow-up periods ranged from 10 days to 61.8 months. Outcome measures included FVC, VC, TLC, DLCO, and FEV1/FVC. The average incidence of adverse events was 26.87% in the treatment group and 27.57% in the control group. Our Data Characteristics Table is presented in Supplementary Table S3.

### Quality assessment of the included studies

**FIGURE 2:**
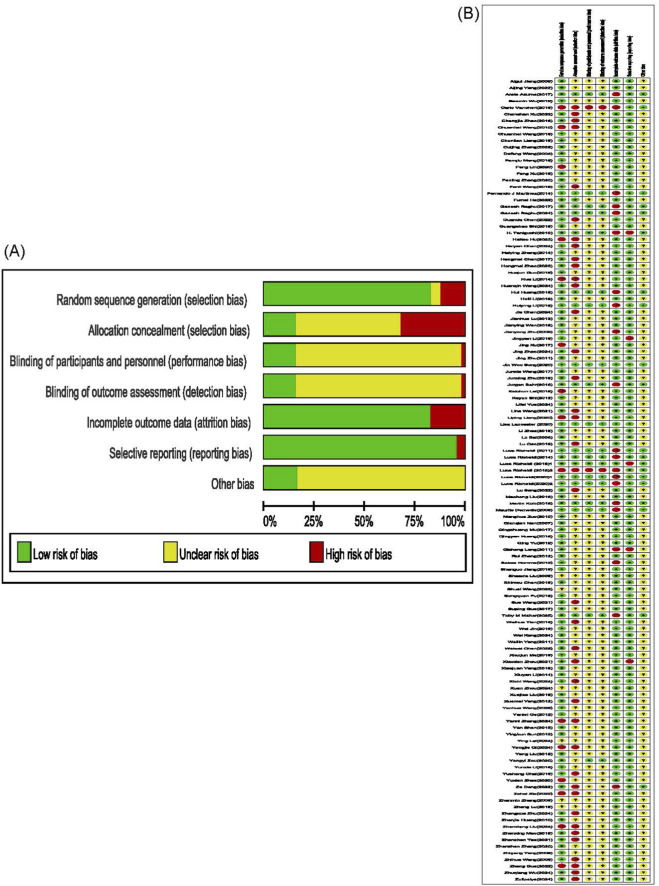
Risk of bias assessment table. **(A)** Risk of bias summary diagram. **(B)** Risk of bias graph diagram.

Using the Cochrane Risk of Bias Assessment Tool, we evaluated the risk of bias and quality of 121 included studies (one consisted of two studies with different risk of bias and quality, thus assessed separately). Regarding random sequence generation, 15 studies used hospital admission numbers or treatment protocols as the basis for allocation and were rated as “high risk”; six studies (Demedts et al., 2005; Zhang et al., 2005; Bai and Wu, 2006; Liu and Qiu, 2006; Wang and Wang, 2006; Wang and Li, 2006) did not explicitly describe how random sequences were generated and were rated as “unclear risk”; the remaining 101 studies employed appropriate randomization methods for allocation and were rated as “low risk.” Regarding allocation concealment, 39 studies (Nan, 2007; Zhigang et al., 2008; Jiang et al., 2009; Zhu et al., 2009; Huang et al., 2010; Taniguchi et al., 2010; Zuo et al., 2010; Long et al., 2011; Richeldi et al., 2011; Yang et al., 2011; Zhu and Chen, 2011; Ge et al., 2012; Homma et al., 2012; Yang et al., 2012; Ying-kun et al., 2012; Yu, 2012; Zhang, 2012; Lu and Gao, 2013; Shi et al., 2013; Li et al., 2014; Li, 2014; Martinez et al., 2014; Richeldi et al., 2014; Zhang, 2014; Chuan-hai et al., 2015; Fu et al., 2015; Huang et al., 2015; Huang, 2015; Jiang, 2015; Jin, 2015; Li et al., 2015; Liu, 2015; Shen et al., 2015; Xian-jun et al., 2015; Zhao, 2015; Behr et al., 2016; Chuanhai and Chenghong, 2016; Li et al., 2016; Liu et al., 2016) were rated “high risk” due to the use of random number tables or pre-disclosure of treatment plans; 19 studies (Richeldi et al., 2011; Liu, 2016; Lu and Fa, 2016; Tian, 2016; Xu et al., 2016; Azuma et al., 2017; Chen and Feng, 2017; Guo, 2017; Mu et al., 2017; Raghu et al., 2017a; Wang, 2017; Xu, 2017; Chai, 2018; Kai-chun et al., 2018; Kolb et al., 2018; Li H., 2018; Li J., 2018; Mao, 2018; Meng, 2018) were rated “low risk” because neither investigators nor participants could predict group assignment before the trial. The remaining 64 studies were rated “unclear risk” owing to unclear allocation methods. Regarding blinding of participants and personnel, as well as outcome assessment, 19 studies (Richeldi et al., 2011; Liu, 2016; Lu and Fa, 2016; Tian, 2016; Xu et al., 2016; Azuma et al., 2017; Chen and Feng, 2017; Guo, 2017; Mu et al., 2017; Raghu et al., 2017a; Wang, 2017; Xu, 2017; Chai, 2018; Kai-chun et al., 2018; Kolb et al., 2018; Li H., 2018; Mao, 2018; Meng, 2018; R et al., 2018) implemented blinding for patients, investigators, and outcome assessors, rated as “low risk”; two studies (Richeldi et al., 2011; Yang et al., 2011) explicitly stated that no blinding was used and were rated “high risk”; the remaining 101 studies had an unclear blinding status and were rated as “unclear risk.” Concerning incomplete outcome data, 21 studies (Richeldi et al., 2011; Yang et al., 2011; Li, 2014; Liu, 2016; Xu, 2017; Kai-chun et al., 2018; Kolb et al., 2018; Mao, 2018; Meng, 2018; Shi et al., 2018; Vancheri et al., 2018; Cao et al., 2018) experienced loss to follow-up for final outcome data collection and were rated “high risk”; the remaining 101 studies had no loss to follow-up and were rated “low risk.” Regarding selective reporting, four studies (Richeldi et al., 2011; Shi et al., 2013; Vancheri et al., 2018; Chen and Guo, 2019) omitted pre-specified methodological reporting items in their final reports and were rated “high risk”; the remaining 118 studies reported comprehensively and were rated “low risk.” For other bias, 19 studies (Richeldi et al., 2011; Yang et al., 2011; Li, 2014; Liu, 2016; Lu and Fa, 2016; Tian, 2016; Xu et al., 2016; Azuma et al., 2017; Chen and Feng, 2017; Guo, 2017; Mu et al., 2017; Raghu et al., 2017a; Wang, 2017; Xu, 2017; Kai-chun et al., 2018; Kolb et al., 2018; Li H., 2018; Meng, 2018) reported no other sources of bias and were rated as “low risk”; the remaining 103 studies were unclear on the presence of other bias sources and were rated as “risk unclear.” In summary, the body of evidence is characterized by a strong performance in random sequence generation (101/122 studies with low risk) and low risk in reporting and attrition biases. However, the credibility of the findings is tempered by a high prevalence of concerns regarding allocation concealment (only 19/122 studies with low risk) and blinding (only 19/122 studies with low risk). The Risk of bias summary diagram is presented in Figure 2A. The Risk of bias graph diagram is presented in Figure 2B.

### Results of Bayesian network meta-analysis and ranking

#### Static lung volume parameters

##### FVC

**FIGURE 3:**
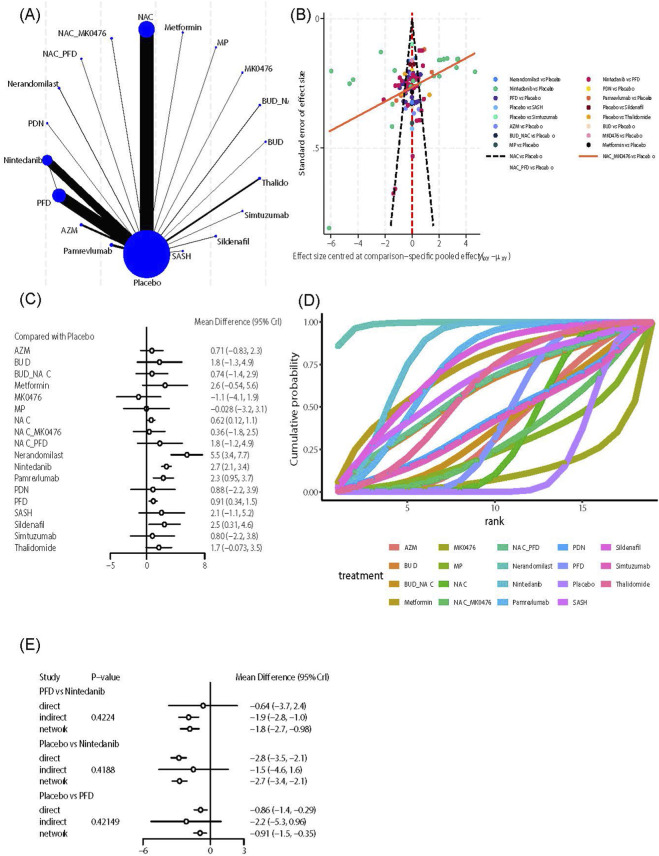
Effects of pharmacological treatments on FVC in idiopathic pulmonary fibrosis. **(A)** Network analysis of evidence. **(B)** The funnel plot. **(C)** The forest plot. **(D)** The SUCRA ranking plot. **(E)** Local inconsistency test diagram.

This outcome indicator includes 19 intervention measures and 116 studies. An evidence network analysis (Figure 3A) showed that NAC had the most direct comparisons with the control group (n = 37), while Budesonide (BUD), Metformin, Montelukast sodium (MK0476), Methylprednisolone (MP), NAC combined with PFD, Prednisone (PDN), Heparin (SASH), and Simtuzumab had limited direct evidence with the control group, and PFD had limited direct evidence with Nintedanib (n = 1). The funnel plot (Figure 3B) is generally symmetrical, but a few scattered points are at the bottom, indicating small studies on NAC. Further effect size analysis shows that, compared with the control group (Figure 3C), NAC (MD = 0.62, 95% CrI [0.12, 1.1]), Nerandomilast (MD = 5.5, 95% CrI [3.4, 7.7]), Nintedanib (MD = 2.7, 95% CrI [2.1, 3.4]), Pamrevlumab (MD = 2.3, 95% CrI [0.95, 3.7]), PFD (MD = 0.91, 95% CrI [0.34, 1.5]), and Sildenafil (MD = 2.5, 95% CrI [0.31, 4.6]) significantly improved FVC. The league table (Supplementary Table S4A) displays comparisons between different interventions, with Nerandomilast showing superiority over AZM (MD = 4.83, 95% CrI [2.19, 7.47]), BUD_NAC (MD = 4.8, 95% CrI [1.74, 7.86]), MK0476 (MD = 6.65, 95% CrI [2.93, 10.37]), MP (MD = 5.57, 95% CrI [1.78, 9.37]), NAC (MD = 4.92, 95% CrI [2.72, 7.12]), NAC_MK0476 (MD = 5.18, 95% CrI [2.14, 8.23]), Nintedanib (MD = 2.81, 95% CrI [0.56, 5.05]), Pamrevlumab (MD = 3.23, 95% CrI [0.69, 5.77]), PDN (MD = 4.67, 95% CrI [0.93, 8.39]), PFD (MD = 4.64, 95% CrI [2.42, 6.85]), Sildenafil (MD = 3.08, 95% CrI [0.05, 6.12]), Simtuzumab (MD = 4.75, 95% CrI [1.02, 8.47]), and Thalidomide (MD = 3.85, 95% CrI [1.07, 6.63]) are more effective at improving FVC; Nintedanib is more effective than AZM (MD = 2.02, 95% CrI [0.35, 3.7]), MK0476 (MD = 3.84, 95% CrI [0.74, 6.96]), NAC (MD = 2.12, 95% CrI [1.29, 2.94]), NAC_MK0476 (MD = 2.38, 95% CrI [0.11, 4.65]), and PFD (MD = 1.83, 95% CrI [0.99, 2.68]) in improving FVC. Regarding efficacy ranking, the SUCRA value and cumulative probability curve (Figure 3D) indicate that Nerandomilast (SUCRA: 98.85%) is the best option, followed by Nintedanib (SUCRA: 80.39%) and Sildenafil (SUCRA: 72.45%). Due to the presence of closed loops, robustness testing was conducted using the node partitioning method, yet no significant local inconsistencies were detected (*P* > 0.05) (Figure 3E).

##### VC

**FIGURE 4:**
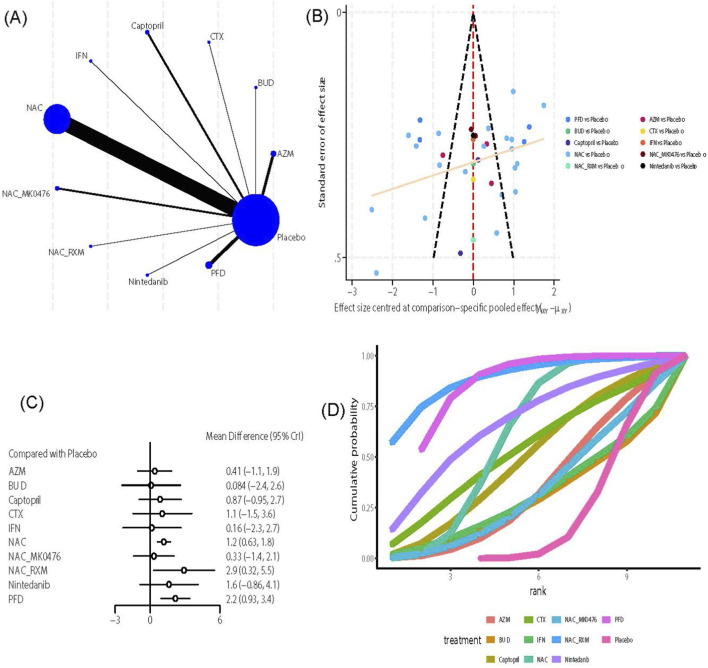
Effects of pharmacological treatments on VC in Idiopathic Pulmonary Fibrosis. **(A)** Network analysis of evidence. **(B)** The funnel plot. **(C)** The forest plot. **(D)** The SUCRA ranking plot.

This outcome indicator included 10 intervention measures and 36 studies (Demedts et al., 2005; Nan, 2007; Zhu et al., 2009; Huang et al., 2010; Taniguchi et al., 2010; Martinez et al., 2014; Li et al., 2015; Liu, 2016; Lu and Fa, 2016; Shi et al., 2018; Chen and Guo, 2019; Guo and Wang, 2019; Jianying et al., 2019; Liang et al., 2019; Wang et al., 2019; Wu and Pan, 2019; Yang and Ren, 2019; Zhao and Xu, 2019; Zhu and Zhu, 2019; Lancaster et al., 2020; Liang, 2020; Lin, 2020; Richeldi et al., 2020a; Richeldi et al., 2020b; Song et al., 2020; Zou and Yin, 2020; Zhang and Chen, 2020; Zhang and Liu, 2020; Zhao, 2020). Network analysis of evidence (Figure 4A) revealed that NAC formed the most extensive direct comparisons with the control group (n = 20). However, direct evidence for BUD, Cyclophosphamide (CTX), IFN-γ1b (IFN), NAC_Roxithromycin (RXM), and Nintedanib showed limited direct evidence against the control group (n = 1). The funnel plot (Figure 4B) exhibited overall symmetry, although a small number of scattered points at the bottom indicated the presence of small-sample studies for NAC. Further effect size analysis indicated that, compared with the control group (Figure 4C), NAC (MD = 1.2, 95% CrI [0.63, 1.8]), NAC_RXM (MD = 2.9, 95% CrI [0.32, 5.5]), and PFD (MD = 2.2, 95% CrI [0.93, 3.4]) significantly improved VC. The league table (Supplementary Table S4B) showed no significant differences between pairwise interventions for improving VC. Regarding treatment ranking, both the SUCRA value and cumulative probability curve (Figure 4D) suggested that NAC_RXM (SUCRA: 88.8%) was the best regimen, followed by PFD (SUCRA: 83.31%) and NAC (SUCRA: 59.87%). Due to the absence of a closed loop, no inconsistency checks were performed.

##### TLC

**FIGURE 5:**
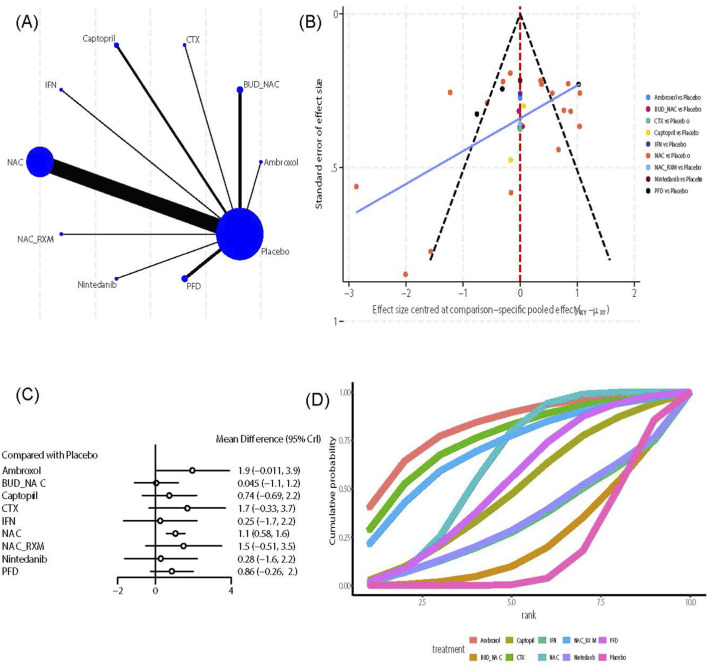
Effects of pharmacological treatments on TLC in idiopathic pulmonary fibrosis. **(A)** Network analysis of evidence. **(B)** The funnel plot. **(C)** The forest plot. **(D)** The SUCRA ranking plot.

This outcome measure included 10 intervention measures and 30 studies (Nan, 2007; Huang et al., 2010; Taniguchi et al., 2010; Ying-kun et al., 2012; Martinez et al., 2014; Jiang, 2015; Jin, 2015; Li et al., 2015; Zhao, 2015; Guo, 2017; Shi et al., 2018; Wang et al., 2019; Wu and Pan, 2019; Yang and Ren, 2019; Zhao and Xu, 2019; Zhu and Zhu, 2019; Zou and Yin, 2020; Zhang and Liu, 2020; Su-e W, 2021; Tao, 2021; Wang et al., 2021; Zhou et al., 2021; Chen and Yin, 2022; Deng, 2022). Network analysis of evidence (Figure 5A) showed that N-acetylcysteine had the most direct comparisons with the control group (n = 17), while direct evidence for Ambroxol, CTX, IFN, NAC_RXM, and Nintedanib versus the control group was limited (n = 1). The funnel plot (Figure 5B) generally appeared symmetrical, although a few scattered points at the bottom indicated small-sample studies on NAC. Further analysis of effect size revealed that NAC (MD = 1.1, 95% CrI [0.58, 1.6]) significantly improved TLC compared to the control group (Figure 5C). The league table (Supplementary Table S4C) indicates no significant differences between pairwise interventions for improving VC. Regarding treatment ranking, both the SUCRA value and the cumulative probability curve (Figure 5D) show that Ambroxol (SUCRA: 82.52%) is the best option, followed by CTX (SUCRA: 76.07%) and NAC_RXM (SUCRA: 70.78%). Due to the absence of a closed loop, no inconsistency checks were performed.

#### Gas exchange capacity indicators

##### DLCO

**FIGURE 6:**
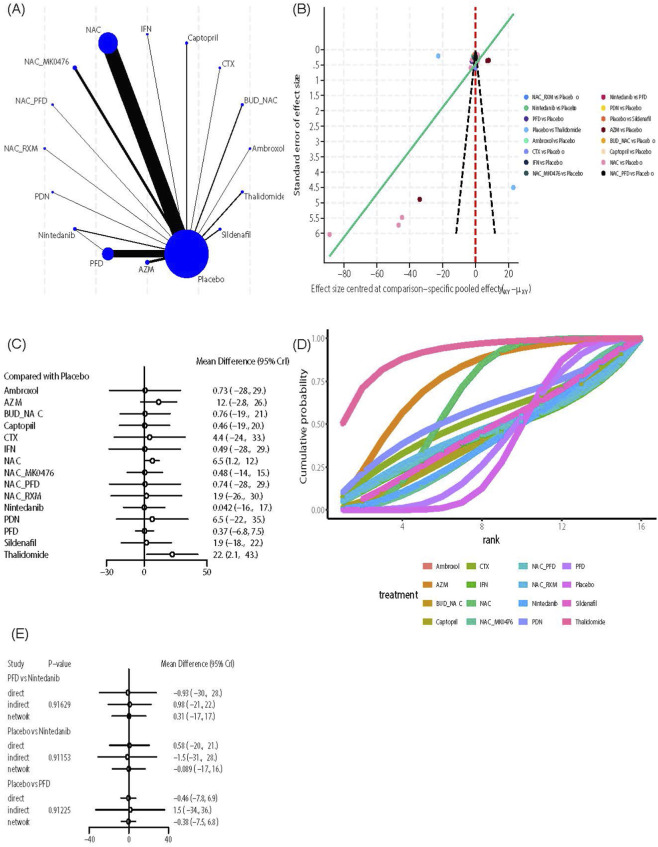
Effects of pharmacological treatments on DLCO in idiopathic pulmonary fibrosis. **(A)** Network analysis of evidence. **(B)** The funnel plot. **(C)** The forest plot. **(D)** The SUCRA ranking plot. **(E)** Local inconsistency test diagram.

This outcome indicator included 16 intervention measures and 68 studies. Evidence network analysis showed that NAC (Figure 6A) had the most extensive direct comparisons with the control group (n = 28), while direct evidence for Ambroxol, CTX, IFN, NAC_PFD, NAC_RXM, PDN, and the control group, as well as for PFD versus Nintedanib, was limited (n = 1). The funnel plot appeared mostly symmetrical (Figure 6B), but a few scattered points at the bottom indicated the presence of small-sample studies for NAC. Further effect size analysis revealed that, compared with the control group (Figure 6C), NAC (MD = 6.5, 95% CrI [1.2, 12]) and Thalidomide (MD = 22, 95% CrI [2.1, 43]) significantly improved DLCO. The league table (Supplementary Table S4D) comparison between interventions showed Thalidomide outperformed PFD (MD = 22.09, 95% CrI [0.6, 43.78]) in improving DLCO, while Nintedanib and PFD showed no significant difference. Regarding treatment ranking, both the SUCRA score and cumulative probability curve indicated (Figure 6D) that thalidomide (SUCRA: 90.93%) was the top-ranked treatment, followed by AZM (SUCRA: 74.90%) and NAC (SUCRA: 63.74%). Due to the presence of closed loops, robustness testing was conducted using the node partitioning method, yet no significant local inconsistencies were detected (*P* > 0.05) (Figure 6E).

#### Dynamic ventilation function indicators

##### FEV1/FVC

**FIGURE 7:**
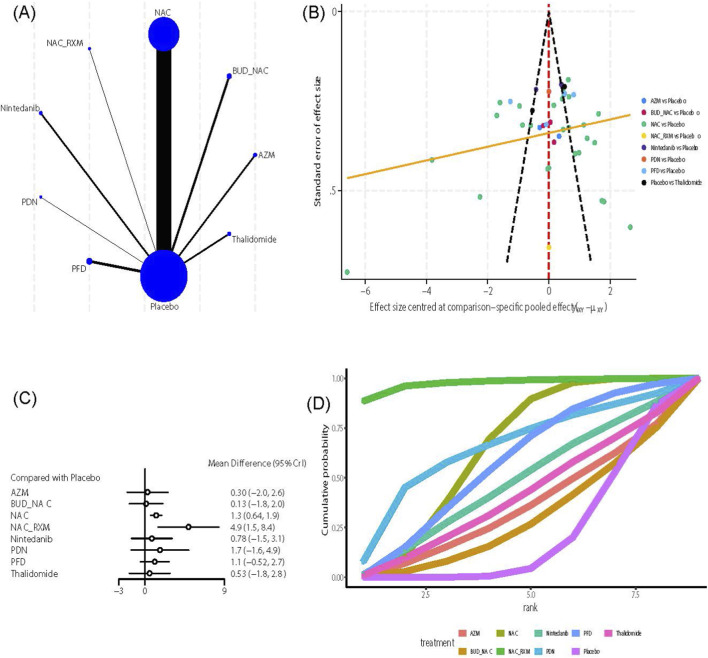
Effects of pharmacological treatments on FEV1/FVC in idiopathic pulmonary fibrosis. **(A)** Network analysis of evidence. **(B)** The funnel plot. **(C)** The forest plot. **(D)** The SUCRA ranking plot.

This outcome indicator included nine intervention measures and 42 studies (Zhang et al., 2005; Nan, 2007; Zhigang et al., 2008; Huang et al., 2010; Zuo et al., 2010; Long et al., 2011; Yang et al., 2011; Zhang, 2012; Shi et al., 2013; Li et al., 2014; Zhang, 2014; Huang, 2015; Jiang, 2015; Jin, 2015; Xian-jun et al., 2015; Zhao, 2015; Li et al., 2016; Liu, 2016; Tian, 2016; Chen and Feng, 2017; Mu et al., 2017; Li H., 2018; Shi et al., 2018; Chen and Guo, 2019; Lin, 2020; Zhang and Chen, 2020; Guo, 2022; Song et al., 2022; Chanchan, 2023; Wang, 2023; Chen, 2024; Kong, 2024; Lei and Liu, 2024; Zhao and Luo, 2024). Evidence network analysis (Figure 7A) showed that NAC had the most extensive direct comparisons with the control group (n = 27), whereas direct evidence for NAC_RXM and PDN versus the control group was limited (n = 1). The funnel plot (Figure 7B) showed overall symmetry, although a few outliers remained at the bottom, indicating the presence of small-sample studies for NAC. Further analysis of effect sizes showed that, compared to the control group (Figure 7C), NAC (MD = 1.3, 95% CrI [0.64, 1.9]) and NAC_RXM (MD = 4.9, 95% CrI [1.5, 8.4]) significantly improved FEV1/FVC. The league table (Supplementary Table S4E) highlights comparisons between the two interventions: NAC_RXM demonstrated greater improvement than AZM (MD = 4.64, 95% CrI [0.45, 8.82]), BUD_NAC (MD = 4.9, 95% CrI [0.87, 8.77]), NAC (MD = 3.68, 95% CrI [0.15, 7.2]), PDN (MD = 3.84, 95% CrI [0.01, 7.66]), and Thalidomide (MD = 4.41, 95% CrI [0.25, 8.57]) in improving FEV1/FVC. In terms of treatment ranking, both the SUCRA value and the cumulative probability curve (Figure 7D) indicated that NAC_RXM (SUCRA: 97.45%) was the best regimen, followed by PDN (SUCRA: 64.18%) and NAC (SUCRA: 63.21%). Due to the absence of a closed loop, no inconsistency checks were performed.

### Effect modifiers analysis for lung function outcomes

#### Baseline age

In the regression analysis, we evaluated how patients’ baseline age (in years) influenced treatment effect measures for five pulmonary function outcomes (FVC, VC, TLC, DLCO, FEV1/FVC). The results are as follows (β represents the regression coefficient, 95% CI shows the 95% confidence interval): FVC: β = −2.35, 95% CI: (−33.56, 19.84); VC: β = −0.93, 95% CI: (−1.68, 0.01); TLC: β = −0.71, 95% CI: (−1.60, 0.17); DLCO: β = −75.25, 95% CI: (−402.21, 177.47); FEV1/FVC: β = −0.23, 95% CI: (−1.78, 2.32). The analysis indicated that age regression coefficients for all outcome measures did not reach statistical significance (P > 0.05). All confidence intervals included zero, suggesting there is insufficient evidence to confirm a significant effect of baseline age on treatment outcomes. Therefore, despite the 40-year age range in the study population, this study found no evidence that baseline age systematically impacts the effectiveness of treatment on pulmonary function outcomes.

#### Follow-up duration

In the regression analysis, we evaluated the effect of patient follow-up duration (in weeks) on treatment outcomes for five pulmonary function measures (FVC, VC, TLC, DLCO, FEV1/FVC). The regression results are as follows (β represents the regression coefficient, and 95% CI indicates the 95% confidence interval): FVC: β = 13.29, 95% CI: (−0.70, 32.02); VC: β = 0.01, 95% CI: (−1.15, 0.83); TLC: β = −0.31, 95% CI: (−1.32, 0.77); DLCO: β = 96.44, 95% CI: (−63.31, 285.56); FEV1/FVC: β = −4.83, 95% CI: (−19.73, 1.35). The analysis showed that the regression coefficients for age across all outcome measures did not reach statistical significance (P > 0.05). All confidence intervals included zero, indicating there is not enough evidence to suggest that follow-up duration has a significant effect on treatment outcomes. Therefore, despite the wide range of follow-up durations in the study population (from 10 days to 61.8 months), this study found no evidence that follow-up duration systematically affected the efficacy of drug therapy on pulmonary function outcomes.

#### Safety

Adverse event information was reported in 75 studies, enabling an evaluation of the safety features of 17 different pharmacological interventions for IPF. The spectrum of reported adverse reactions was predominantly characterized by gastrointestinal disturbances, respiratory infections, hepatic function test abnormalities, and dermatological events. A comparative analysis of adverse drug reaction profiles revealed notable differences among the key therapeutic agents.

Gastrointestinal events, particularly diarrhea, were frequently associated with antifibrotic agents. Nintedanib treatment was linked to a substantial incidence of diarrhea, as evidenced by reports of 190 cases in one study versus 38 in the placebo group. A similar trend, though less pronounced, was observed with pirfenidone. Nausea and vomiting were common adverse effects recorded in studies investigating NAC, both as monotherapy and in combination regimens. Respiratory tract infections, including upper respiratory infections, were reported across multiple drug classes. For instance, azithromycin and nintedanib studies documented a higher frequency of such events compared to their respective placebo controls.

Hepatic safety emerged as an important consideration, with elevations in liver enzymes being reported for both pirfenidone and nintedanib. Dermatological reactions, most notably photosensitivity, were a distinctive adverse effect associated with pirfenidone use. Other adverse events, such as hypertension and blood glucose dysregulation, were reported infrequently and without a clear pattern of association with any single intervention.

The majority of these adverse events were graded as mild to moderate in severity. Management typically involved symptomatic treatment or temporary drug discontinuation, with events generally resolving without sequelae. Critically, no deaths were directly attributed to any of the study medications. It is important to note that significant heterogeneity in the reporting methodologies and grading scales for adverse events across the included studies precluded a formal quantitative meta-analysis of safety data. A detailed tabular summary of adverse reactions stratified by intervention is provided in Supplementary Table S5.

## Discussions

IPF is a chronic, progressive fibrotic interstitial lung disease characterised by ongoing scarring of the pulmonary interstitium (Somogyi et al., 2019). Recently, PFD and nintedanib have become common treatments for IPF (Karimi-Shah and Chowdhury, 2015), effectively slowing lung function decline and delaying disease progression (Biondini et al., 2020). However, long-term use can cause severe gastrointestinal reactions and liver damage (Kou et al., 2024). Therefore, finding IPF treatments with better efficacy and safety is essential. Network meta-analysis (NMA) is a valuable method for combining clinical research evidence and comparing the effectiveness of different interventions (Brignardello-Petersen and Guyatt, 2025). Many randomised controlled trials (RCTs) have tested various drugs on IPF, with lung function as a key measure of treatment success. Still, direct comparisons between different drugs are limited, and reported lung function metrics are often isolated, lacking systematic integration of outcomes from various pharmaceutical approaches. This study uses NMA to systematically combine existing RCT evidence, directly comparing the relative effectiveness of different IPF drug regimens on important pulmonary function indicators (FVC, VC, TLC, DLCO, FEV1/FVC). This approach not only fills evidence gaps and improves evidence quality but also highlights the strengths and weaknesses of different drugs in improving various lung function metrics. As a result, it helps guide precise clinical medication choices and optimise personalised treatment strategies for IPF patients.

### Summary of results

To our knowledge, no previous NMA has investigated the effects of different types of drugs on pulmonary function indicators. This analysis included 20 drugs: Ambroxol, Azithromycin (AZM), Budesonide (BUD), Captopril, Cyclophosphamide (CTX), Heparin (SASH), IFN-γ1b (IFN), Metformin, Methylprednisolone (MP), Montelukast sodium (MK0476), N-acetylcysteine (NAC), Nerandomilast, Nintedanib, Pamrevlumab, Pirfenidone (PFD), Prednisone (PDN), Roxithromycin (RXM), Sildenafil, Simtuzumab, and Thalidomide. The top three most frequently appearing drugs were NAC, PFD, and Nintedanib, indicating that the drugs included in the analysis reflect actual clinical practice. PFD and Nintedanib are widely used as first-line antifibrotic drugs for IPF, and their high frequency of appearance aligns with expectations. The relatively frequent use of NAC for IPF treatment may relate to its multiple pharmacological mechanisms, including antioxidant (Raghu et al., 2021), expectorant (Ohnishi et al., 2024), and anti-inflammatory effects (Mamashli et al., 2022), its potential therapeutic benefits (Podolanczuk et al., 2021; Raghu et al., 2017b) and favourable safety profile (Calverley et al., 2021) are common used in combination therapy regimens (Velez and Nambiar, 2015). Among the five pulmonary function outcome measures, although some interventions did not show significant superiority over the control group, cumulative ordered probability (SUCRA) analysis indicated that all interventions collectively demonstrated greater efficacy compared to the control group. Specifically, Nerandomilast was the most effective for improving FVC, NAC combined with RXM was most effective for enhancing VC and FEV1/FVC, Ambroxol was the best for improving TLC, and Thalidomide was the most effective for enhancing DLCO. Regression analysis showed that baseline age and follow-up duration did not significantly impact efficacy (*P* > 0.05), supporting the robustness of the results.

### Mechanisms of optimal therapies

#### Nerandomilast improves FVC: multilevel effects targeting PDE4B to modulate fibrosis-related pathways

Nerandomilast, a novel phosphodiesterase-4B (PDE4B) inhibitor, has shown potential in the IPF treatment landscape over the past 2 years. It significantly improves FVC in IPF patients through a multi-level synergistic mechanism (Richeldi et al., 2025). At the molecular level, Nerandomilast increases cAMP levels (Insel et al., 2012), interferes with TGF-β (Liu et al., 2025), and disrupts GPCR signaling pathways (Haak et al., 2020). Simultaneously, it reduces p38 MAPK phosphorylation, disrupts MAPK pathways, and decreases the activity of key pro-fibrotic drivers (Reininger et al., 2025). At the cellular level, Nerandomilast not only lowers pro-fibrotic factor secretion and inhibits the transformation of pulmonary fibroblasts into myofibroblasts (Reininger et al., 2024) but also modulates inflammatory cell function to lessen persistent inflammatory stimulation (Castelino and Adegunsoye, 2025). At the tissue level, Nerandomilast significantly decreases excessive deposition of ECM components such as collagen and fibronectin in lung tissue (Insel et al., 2012), while also helping to slow alveolar structural destruction and progressive interstitial thickening (Kolb et al., 2023). During clinical use, mild gastrointestinal side effects like nausea and vomiting may occur, which usually resolve on their own. If symptoms worsen, symptomatic treatment with gastric mucosal protectants is recommended (Robichaud et al., 2002).

#### NAC combined with RXM improves VC and FEV1/FVC: dual pulmonary function optimisation targeting the oxidative stress-inflammation

##### Improves pulmonary parenchymal fibrosis and enhances VC

The main pathological feature of IPF is decreased lung tissue elasticity and compliance caused by progressive pulmonary fibrosis (Richeldi et al., 2012; Plantier et al., 2018). NAC slows fibrosis progression by scavenging ROS to reduce oxidative stress damage to alveolar epithelial cells and the pulmonary interstitium (Raghu et al., 2021) and by lowering inflammatory responses (Mamashli et al., 2022). RXM reduces chronic inflammation and its fibrogenic stimulation through inhibiting the NF-κB signalling pathway (Choi et al., 2015), which decreases the accumulation of proinflammatory factors and fibrosis markers (Ozsvari et al., 2018). RXM’s anti-inflammatory and anti-fibrotic effects (Zhang et al., 2021). The combined effect of NAC and RXM effectively slowed pulmonary fibrosis progression, preserved pulmonary tissue elasticity, promoted lung expansion, and consequently improved VC.

##### Alleviate small airway dysfunction and optimise FEV1/FVC ratio

Small airway involvement often accompanies IPF, presenting as inflammatory infiltration and mucus plug formation (Ikezoe et al., 2021). RXM, a potent anti-inflammatory macrolide antibiotic (Kelly et al., 2018), inhibits the NF-κB signalling pathway, targeting small airways to reduce neutrophil infiltration (Choi et al., 2015) and airway resistance. NAC lessens local oxidative damage by scavenging ROS (Raghu et al., 2021) and its secondary inflammatory response (Mamashli et al., 2022), while also breaking mucin disulfide bonds to lower sputum viscosity (Ohnishi et al., 2024). This combination enhances secretion clearance and decreases airway obstruction. Reducing inflammation and oxidative stress helps preserve the structural integrity of small airway epithelial cells, leading to improved expiratory flow dynamics reflected in FEV1, an early indicator of forced expiratory capacity (Sun et al., 2021). When small airway resistance decreases, FEV1 improvements often surpass FVC’s, thus supporting or improving the FEV1/FVC ratio.

The combination of NAC and RXM produces dual effects by targeting the standard core mechanisms that drive IPF from the alveoli to the small airways—oxidative stress and chronic inflammation. These two pathophysiological processes are closely interconnected, and the synergistic action of NAC and RXM operates through multi-targeted effects to simultaneously enhance both VC and FEV1/FVC, two key indicators of lung function.

##### Ambroxol improves TLC: multilevel ventilation optimisation targeting the surfactant-inflammation-cilia

Ambroxol, as a mucokinetic enhancer (Wu et al., 2025), improves pulmonary ventilation through multiple pathways (Zhi et al., 2011). At the molecular level, it stimulates alveolar type II epithelial cells to synthesise and secrete pulmonary surfactant, reducing alveolar surface tension and preventing alveolar collapse (Wirtz, 2000), helping to maintain alveolar volume. Concurrently, it inhibits ROS production (Lee et al., 2002), suppresses proteolytic granule release, and strengthens the endogenous anti-protease barrier (Ottonello et al., 2003), while multi-targetedly suppressing neutrophil activity to alleviate local inflammatory responses (Winsel et al., 1985). At the cellular and tissue levels, these anti-inflammatory effects help reduce inflammatory narrowing of small airways and alveolar wall oedema, thereby improving lung tissue compliance (Chen et al., 2015). Ambroxol also stimulates ciliated epithelial cells, increasing ciliary beating frequency and coordination, significantly enhancing the clearance capacity of the mucociliary clearance system (Disse and Ziegler, 1987), and reducing small airway obstruction. These effects optimise lung expansion capacity, enabling greater gas accommodation in lung tissue during maximum inspiration, ultimately leading to a significant improvement in TLC in IPF patients.

##### Thalidomide improves DLCO: multilevel intervention targeting the immunomodulation-microcirculation

This NMA revealed that thalidomide, an immunomodulatory agent (El-Zahabi et al., 2020), significantly enhances DLCO. It substantially improves pulmonary gas exchange efficiency through a multilevel synergistic mechanism. At the molecular level, thalidomide targets the TNF-α signalling pathway (Majumder et al., 2012), decreasing the expression of pro-inflammatory factors such as IL-1β and IL-6 (Dong et al., 2017). This reduces inflammatory damage and strengthens the integrity of the alveolar-capillary barrier (Sun et al., 2023). Additionally, thalidomide modulates the synthesis and release of vascular endothelial growth factor (VEGF), enhancing pulmonary microvascular endothelial function and lowering diffusion resistance (Huang et al., 2022). At the cellular level, thalidomide inhibits excessive activation of macrophages and neutrophils, reducing inflammatory injury (Kumar et al., 2010). At the same time, it protects alveolar epithelial cells by counteracting oxidative stress and suppressing apoptosis (Dong et al., 2017), helping to maintain the stability of the gas exchange surface. At the tissue level, thalidomide decreases pulmonary interstitial inflammatory infiltration and collagen deposition, alleviating alveolar wall thickening and microvascular occlusion (Li et al., 2022), which lowers gas diffusion resistance. Ultimately, this leads to an improvement in DLCO, a key indicator of pulmonary function.

##### Safety

While comparative efficacy on lung function parameters is a crucial consideration in treatment selection, a thorough evaluation of the safety profiles associated with these interventions is equally indispensable for guiding balanced clinical decisions. The adverse event data synthesized in this analysis reveal that the toxicity characteristics of each drug align closely with their distinct pharmacological mechanisms. For instance, the frequent occurrence of diarrhea with nintedanib is consistent with its inhibition of multiple receptor tyrosine kinases, including the vascular endothelial growth factor receptor pathway, which is known to impact gastrointestinal mucosal integrity (Tu et al., 2024). Likewise, the photosensitivity reactions commonly associated with pirfenidone highlight the importance of strict photoprotective measures for patients receiving this agent (Kou et al., 2024).

In contrast, NAC-based therapies, whether administered as monotherapy or in combination, exhibited a relatively favorable tolerability profile, with gastrointestinal symptoms representing the most frequently reported concerns. This supports the use of NAC, particularly in combination regimens, where a positive benefit-risk balance is a key determinant (Calverley et al., 2021). However, caution is warranted in interpreting these safety findings due to inconsistencies in adverse event reporting across the included studies. Variations in definitions, reporting thresholds, and monitoring protocols introduce considerable uncertainty into cross-trial comparisons.

The absence of standardized, systematic safety reporting in IPF clinical trials constitutes a significant evidence gap. Future research should prioritize the consistent application of established toxicity criteria, such as the Common Terminology Criteria for Adverse Events (CTCAE), to enable more robust cross-comparisons and network meta-analyses of treatment safety. Moreover, long-term safety data extending beyond the typical duration of clinical trials are urgently needed to fully characterize the risks associated with prolonged use of these agents in a progressive disease such as IPF. Therefore, although efficacy remains a primary driver in therapy selection, the choice of treatment for individual IPF patients should be personalized, carefully weighing the potential for functional improvement against the specific adverse event profile, while also considering the patient’s comorbidities and individual risk tolerance.

### Limitations

This study is subject to several inherent limitations. First, considerable clinical and methodological heterogeneity was observed among the included trials, which may compromise the internal validity of the pooled estimates. Variations in participant characteristics—such as age—as well as differences in intervention protocols regarding dosage and treatment duration, divergent study designs with varying levels of methodological rigor, and inconsistencies in outcome measurement approaches all contributed to this heterogeneity. Furthermore, the number of available studies investigating newer agents such as Nerandomilast and certain combination regimens remains limited, constraining the statistical power and reliability of the corresponding analyses.

Second, the overall risk of bias assessment identified specific methodological concerns, particularly regarding allocation concealment and blinding in a considerable proportion of the included studies. These issues may introduce performance and detection bias, suggesting that the estimated treatment effects should be interpreted with appropriate caution.

Third, the evidence network heavily relies on indirect comparisons. The absence of head-to-head trials for several key drug pairs reduces the certainty of the estimated treatment effects. Although the use of a Bayesian framework for network meta-analysis provides a methodological basis for indirect inference (Veroniki et al., 2016), the conclusions drawn still require validation through future high-quality direct-comparison trials.

Fourth, there is evidence of potential publication bias, particularly among smaller studies, as suggested by asymmetry in funnel plots. This pattern indicates a possible underrepresentation of negative or null results, which could lead to overestimation of the treatment benefits associated with more established interventions such as NAC, pirfenidone, and nintedanib, while potentially underestimating the efficacy of newer drugs including Pamrevlumab.

Fifth, the incorporation of studies that required data conversion, such as the transformation of standard error of the mean to standard deviation, or extraction from graphical representations introduces a potential source of measurement imprecision. Although we implemented a rigorous dual investigator extraction and verification process with a prespecified tolerance to minimize errors in data acquired from figures, and the conversion of standard error of the mean to standard deviation is statistically exact, a formal sensitivity analysis excluding these studies was not performed. This was due to the limited number of such studies for each specific outcome. Their exclusion would have compromised the connectivity of the treatment network or substantially reduced statistical power. While the high agreement between independent extractors provides confidence, this factor remains a relevant consideration when interpreting the findings.

Despite these limitations, several methodological safeguards were implemented to enhance the robustness of the findings. Specific measures included: prospective registration of the study protocol on the PROSPERO platform; application of a Bayesian random-effects model to address heterogeneity; regression adjustment for key covariates such as baseline age and follow-up duration; rigorous double-blind data extraction and verification procedures for graphical data; and marking all studies undergoing data transformation or extraction in supplementary data extraction tables to ensure full transparency.

## Conclusion

This NMA is the first comprehensive synthesis of drug therapies’ effects on lung function in IPF. Results show different benefits among drugs for improving specific pulmonary function measures. Due to ongoing methodological limitations and potential publication bias, caution should be used when interpreting and applying these meta-analysis results. Future research should focus on: (1) conducting head-to-head randomised controlled trials comparing drugs; (2) examining dose-response relationships between medication doses and pulmonary function outcomes; and (3) standardising outcome reporting to ensure thorough documentation of pulmonary function, providing a solid evidence base for future studies.

## Data Availability

The original contributions presented in the study are included in the article/Supplementary Material, further inquiries can be directed to the corresponding authors.
