# Supplementary material for: Network meta-analysis of pharmacological treatments for idiopathic pulmonary fibrosis: evaluating effects on lung function
Source: Front Pharmacol. 2026 Feb 23;17:1761899. doi: 10.3389/fphar.2026.1761899 (PMC12968560; doi:10.3389/fphar.2026.1761899)
Supplement: Supplementary file 1 [file Table1.docx]

Supplementary Material

# Supplementary Table S1 PRISMA 2020 checklist

| **Section/Topic** | **Item #** | **Checklist Item** | **Reported on Page #** |
| --- | --- | --- | --- |
| **TITLE** |  |  |  |
| Title | 1 | Identify the report as a systematic review incorporating a network meta-analysis (or related form of meta-analysis). | Title |
|  |  |  |  |
| **ABSTRACT** |  |  |  |
| Structured summary | 2 | Provide a structured summary including, as applicable:  **Background:** main objectives  **Methods:** data sources; study eligibility criteria, participants, and interventions; study appraisal; and synthesis methods, such as network meta-analysis.  **Results:** number of studies and participants identified; summary estimates with corresponding confidence/credible intervals; treatment rankings may also be discussed. Authors may choose to summarize pairwise comparisons against a chosen treatment included in their analyses for brevity.  **Discussion/Conclusions:** limitations; conclusions and implications of findings.  **Other:** primary source of funding; systematic review registration number with registry name. | Abstract |
|  |  |  |  |
| **INTRODUCTION** |  |  |  |
| Rationale | 3 | Describe the rationale for the review in the context of what is already known, including mention of why a network meta-analysis has been conducted. | Introduction First Paragraph |
| Objectives | 4 | Provide an explicit statement of questions being addressed, with reference to participants, interventions, comparisons, outcomes, and study design (PICOS). | Introduction Paragraphs 2 and 3 |
|  |  |  |  |
| **METHODS** |  |  |  |
| Protocol and registration | 5 | Indicate whether a review protocol exists and if and where it can be accessed (e.g., Web address); and, if available, provide registration information, including registration number. | Method First Paragraph |
| Eligibility criteria | 6 | Specify study characteristics (e.g., PICOS, length of follow-up) and report characteristics (e.g., years considered, language, publication status) used as criteria for eligibility, giving rationale. Clearly describe eligible treatments included in the treatment network, and note whether any have been clustered or merged into the same node (with justification). | Methods-Inclusion and exclusion criteria-Table 1 |
| Information sources | 7 | Describe all information sources (e.g., databases with dates of coverage, contact with study authors to identify additional studies) in the search and date last searched. | Methods-Information source and search strategy |
| Search | 8 | Present full electronic search strategy for at least one database, including any limits used, such that it could be repeated. | Methods-Information source and search strategy-Supplementary Table S2 |
| Study selection | 9 | State the process for selecting studies (i.e., screening, eligibility, included in systematic review, and, if applicable, included in the meta-analysis). | Methods-Study selection |
| Data collection process | 10 | Describe method of data extraction from reports (e.g., piloted forms, independently, in duplicate) and any processes for obtaining and confirming data from investigators. | Methods-Extraction and Analysis |
| Data items | 11 | List and define all variables for which data were sought (e.g., PICOS, funding sources) and any assumptions and simplifications made. | Methods-Extraction and Analysis |
| **Geometry of the network** | **S1** | Describe methods used to explore the geometry of the treatment network under study and potential biases related to it. This should include how the evidence base has been graphically summarized for presentation, and what characteristics were compiled and used to describe the evidence base to readers. | Methods-Extraction and Analysis |
| Risk of bias within individual studies | 12 | Describe methods used for assessing risk of bias of individual studies (including specification of whether this was done at the study or outcome level), and how this information is to be used in any data synthesis. | Methods-Data synthesis and analysis |
| Summary measures | 13 | State the principal summary measures (e.g., risk ratio, difference in means). Also describe the use of additional summary measures assessed, such as treatment rankings and surface under the cumulative ranking curve (SUCRA) values, as well as modified approaches used to present summary findings from meta-analyses. | Methods-Data synthesis and analysis |
| Planned methods of analysis | 14 | Describe the methods of handling data and combining results of studies for each network meta-analysis. This should include, but not be limited to:   - Handling of multi-arm trials; - Selection of variance structure; - Selection of prior distributions in Bayesian analyses; and - Assessment of model fit. | Methods-Data synthesis and analysis |
| **Assessment of Inconsistency** | **S2** | Describe the statistical methods used to evaluate the agreement of direct and indirect evidence in the treatment network(s) studied. Describe efforts taken to address its presence when found. | Methods-Data synthesis and analysis |
| Risk of bias across studies | 15 | Specify any assessment of risk of bias that may affect the cumulative evidence (e.g., publication bias, selective reporting within studies). | Methods-Risk of Bias Assessment |
| Additional analyses | 16 | Describe methods of additional analyses if done, indicating which were pre-specified. This may include, but not be limited to, the following:   - Sensitivity or subgroup analyses; - Meta-regression analyses; - Alternative formulations of the treatment network; and - Use of alternative prior distributions for Bayesian analyses (if applicable). | Methods-Data synthesis and analysis |
|  |  |  |  |
| **RESULTS†** |  |  |  |
| Study selection | 17 | Give numbers of studies screened, assessed for eligibility, and included in the review, with reasons for exclusions at each stage, ideally with a flow diagram. | Results-Literature screening process and results |
| **Presentation of network structure** | **S3** | Provide a network graph of the included studies to enable visualization of the geometry of the treatment network. | Results-Figure3A;4A;5A;6A;7A |
| **Summary of network geometry** | **S4** | Provide a brief overview of characteristics of the treatment network. This may include commentary on the abundance of trials and randomized patients for the different interventions and pairwise comparisons in the network, gaps of evidence in the treatment network, and potential biases reflected by the network structure. | Results-Results of Bayesian network meta-analysis and ranking |
| Study characteristics | 18 | For each study, present characteristics for which data were extracted (e.g., study size, PICOS, follow-up period) and provide the citations. | Results-Description of included studies |
| Risk of bias within studies | 19 | Present data on risk of bias of each study and, if available, any outcome level assessment. | Results-Quality assessment of the included studies |
| Results of individual studies | 20 | For all outcomes considered (benefits or harms), present, for each study: 1) simple summary data for each intervention group, and 2) effect estimates and confidence intervals. Modified approaches may be needed to deal with information from larger networks. | Results-Results of Bayesian network meta-analysis and ranking |
| Synthesis of results | 21 | Present results of each meta-analysis done, including confidence/credible intervals. In larger networks, authors may focus on comparisons versus a particular comparator (e.g. placebo or standard care), with full findings presented in an appendix. League tables and forest plots may be considered to summarize pairwise comparisons. If additional summary measures were explored (such as treatment rankings), these should also be presented. | Results-Figure3;4;5;6;7 |
| **Exploration for inconsistency** | **S5** | Describe results from investigations of inconsistency. This may include such information as measures of model fit to compare consistency and inconsistency models, P values from statistical tests, or summary of inconsistency estimates from different parts of the treatment network. | Results-Results of Bayesian network meta-analysis and ranking |
| Risk of bias across studies | 22 | Present results of any assessment of risk of bias across studies for the evidence base being studied. | Results-Results of Bayesian network meta-analysis and ranking |
| Results of additional analyses | 23 | Give results of additional analyses, if done (e.g., sensitivity or subgroup analyses, meta-regression analyses, alternative network geometries studied, alternative choice of prior distributions for Bayesian analyses, and so forth). | Results-Effect Modifiers Analysis for Lung Function Outcomes |
|  |  |  |  |
| **DISCUSSION** |  |  |  |
| Summary of evidence | 24 | Summarize the main findings, including the strength of evidence for each main outcome; consider their relevance to key groups (e.g., healthcare providers, users, and policy-makers). | Discussions-Summary of Results |
| Limitations | 25 | Discuss limitations at study and outcome level (e.g., risk of bias), and at review level (e.g., incomplete retrieval of identified research, reporting bias). Comment on the validity of the assumptions, such as transitivity and consistency. Comment on any concerns regarding network geometry (e.g., avoidance of certain comparisons). | Discussions-Limitations |
| Conclusions | 26 | Provide a general interpretation of the results in the context of other evidence, and implications for future research. | Conclusion |
|  |  |  |  |
| **FUNDING** |  |  |  |
| Funding | 27 | Describe sources of funding for the systematic review and other support (e.g., supply of data); role of funders for the systematic review. This should also include information regarding whether funding has been received from manufacturers of treatments in the network and/or whether some of the authors are content experts with professional conflicts of interest that could affect use of treatments in the network. | Funding |

# Supplementary Table S2 A detailed search strategy for each database

| **PubMed**   \| Search number \| Query \| Results \| \| --- \| --- \| --- \| \| 12 \| ((((("Idiopathic Pulmonary Fibrosis"[Mesh]) OR (((((((Idiopathic Pulmonary Fibrosis[Title/Abstract]) OR (Idiopathic Pulmonary Fibroses[Title/Abstract])) OR (Pulmonary Fibroses, Idiopathic[Title/Abstract])) OR (Pulmonary Fibrosis, Idiopathic[Title/Abstract])) OR (Idiopathic Fibrosing Alveolitis, Chronic Form[Title/Abstract])) OR (Familial Idiopathic Pulmonary Fibrosis[Title/Abstract])) OR (Idiopathic Pulmonary Fibrosis, Familial[Title/Abstract]))) OR ("Pulmonary Fibrosis"[Mesh])) OR (((((((((Pulmonary Fibrosis[Title/Abstract]) OR (Fibroses, Pulmonary[Title/Abstract])) OR (Fibrosis, Pulmonary[Title/Abstract])) OR (Pulmonary Fibroses[Title/Abstract])) OR (Alveolitis, Fibrosing[Title/Abstract])) OR (Alveolitides, Fibrosing[Title/Abstract])) OR (Fibrosing Alveolitides[Title/Abstract])) OR (Fibrosing Alveolitis[Title/Abstract])) OR (Idiopathic Diffuse Interstitial Pulmonary Fibrosis[Title/Abstract]))) AND ((("Drug Therapy"[Mesh]) OR ((((((Drug therapy[Title/Abstract]) OR (Pharmacotherapy[Title/Abstract])) OR (Pharmacotherapies[Title/Abstract])) OR (Therapy, Drug[Title/Abstract])) OR (Drug Therapies[Title/Abstract])) OR (Therapies, Drug[Title/Abstract]))) OR ((((((((pirfenidone[Title/Abstract]) OR (nintedanib[Title/Abstract])) OR (Acetylcysteine[Title/Abstract])) OR (colchicine[Title/Abstract])) OR (endothelin receptor antagonist[Title/Abstract])) OR (simtuzumab[Title/Abstract])) OR (imatinib[Title/Abstract])) OR (warfarin[Title/Abstract])))) AND (randomized controlled trial[Publication Type] OR randomized[Title/Abstract] OR placebo[Title/Abstract]) \| 891 \| \| 11 \| randomized controlled trial[Publication Type] OR randomized[Title/Abstract] OR placebo[Title/Abstract] \| 1,127,447 \| \| 10 \| (((("Idiopathic Pulmonary Fibrosis"[Mesh]) OR (((((((Idiopathic Pulmonary Fibrosis[Title/Abstract]) OR (Idiopathic Pulmonary Fibroses[Title/Abstract])) OR (Pulmonary Fibroses, Idiopathic[Title/Abstract])) OR (Pulmonary Fibrosis, Idiopathic[Title/Abstract])) OR (Idiopathic Fibrosing Alveolitis, Chronic Form[Title/Abstract])) OR (Familial Idiopathic Pulmonary Fibrosis[Title/Abstract])) OR (Idiopathic Pulmonary Fibrosis, Familial[Title/Abstract]))) OR ("Pulmonary Fibrosis"[Mesh])) OR (((((((((Pulmonary Fibrosis[Title/Abstract]) OR (Fibroses, Pulmonary[Title/Abstract])) OR (Fibrosis, Pulmonary[Title/Abstract])) OR (Pulmonary Fibroses[Title/Abstract])) OR (Alveolitis, Fibrosing[Title/Abstract])) OR (Alveolitides, Fibrosing[Title/Abstract])) OR (Fibrosing Alveolitides[Title/Abstract])) OR (Fibrosing Alveolitis[Title/Abstract])) OR (Idiopathic Diffuse Interstitial Pulmonary Fibrosis[Title/Abstract]))) AND ((("Drug Therapy"[Mesh]) OR ((((((Drug therapy[Title/Abstract]) OR (Pharmacotherapy[Title/Abstract])) OR (Pharmacotherapies[Title/Abstract])) OR (Therapy, Drug[Title/Abstract])) OR (Drug Therapies[Title/Abstract])) OR (Therapies, Drug[Title/Abstract]))) OR ((((((((pirfenidone[Title/Abstract]) OR (nintedanib[Title/Abstract])) OR (Acetylcysteine[Title/Abstract])) OR (colchicine[Title/Abstract])) OR (endothelin receptor antagonist[Title/Abstract])) OR (simtuzumab[Title/Abstract])) OR (imatinib[Title/Abstract])) OR (warfarin[Title/Abstract]))) \| 6,185 \| \| 9 \| (("Drug Therapy"[Mesh]) OR ((((((Drug therapy[Title/Abstract]) OR (Pharmacotherapy[Title/Abstract])) OR (Pharmacotherapies[Title/Abstract])) OR (Therapy, Drug[Title/Abstract])) OR (Drug Therapies[Title/Abstract])) OR (Therapies, Drug[Title/Abstract]))) OR ((((((((pirfenidone[Title/Abstract]) OR (nintedanib[Title/Abstract])) OR (Acetylcysteine[Title/Abstract])) OR (colchicine[Title/Abstract])) OR (endothelin receptor antagonist[Title/Abstract])) OR (simtuzumab[Title/Abstract])) OR (imatinib[Title/Abstract])) OR (warfarin[Title/Abstract])) \| 1,713,669 \| \| 8 \| (((((((pirfenidone[Title/Abstract]) OR (nintedanib[Title/Abstract])) OR (Acetylcysteine[Title/Abstract])) OR (colchicine[Title/Abstract])) OR (endothelin receptor antagonist[Title/Abstract])) OR (simtuzumab[Title/Abstract])) OR (imatinib[Title/Abstract])) OR (warfarin[Title/Abstract]) \| 85,219 \| \| 7 \| (((((Drug therapy[Title/Abstract]) OR (Pharmacotherapy[Title/Abstract])) OR (Pharmacotherapies[Title/Abstract])) OR (Therapy, Drug[Title/Abstract])) OR (Drug Therapies[Title/Abstract])) OR (Therapies, Drug[Title/Abstract]) \| 108,355 \| \| 6 \| "Drug Therapy"[Mesh] \| 1,561,236 \| \| 5 \| ((("Idiopathic Pulmonary Fibrosis"[Mesh]) OR (((((((Idiopathic Pulmonary Fibrosis[Title/Abstract]) OR (Idiopathic Pulmonary Fibroses[Title/Abstract])) OR (Pulmonary Fibroses, Idiopathic[Title/Abstract])) OR (Pulmonary Fibrosis, Idiopathic[Title/Abstract])) OR (Idiopathic Fibrosing Alveolitis, Chronic Form[Title/Abstract])) OR (Familial Idiopathic Pulmonary Fibrosis[Title/Abstract])) OR (Idiopathic Pulmonary Fibrosis, Familial[Title/Abstract]))) OR ("Pulmonary Fibrosis"[Mesh])) OR (((((((((Pulmonary Fibrosis[Title/Abstract]) OR (Fibroses, Pulmonary[Title/Abstract])) OR (Fibrosis, Pulmonary[Title/Abstract])) OR (Pulmonary Fibroses[Title/Abstract])) OR (Alveolitis, Fibrosing[Title/Abstract])) OR (Alveolitides, Fibrosing[Title/Abstract])) OR (Fibrosing Alveolitides[Title/Abstract])) OR (Fibrosing Alveolitis[Title/Abstract])) OR (Idiopathic Diffuse Interstitial Pulmonary Fibrosis[Title/Abstract])) \| 63,365 \| \| 4 \| ((((((((Pulmonary Fibrosis[Title/Abstract]) OR (Fibroses, Pulmonary[Title/Abstract])) OR (Fibrosis, Pulmonary[Title/Abstract])) OR (Pulmonary Fibroses[Title/Abstract])) OR (Alveolitis, Fibrosing[Title/Abstract])) OR (Alveolitides, Fibrosing[Title/Abstract])) OR (Fibrosing Alveolitides[Title/Abstract])) OR (Fibrosing Alveolitis[Title/Abstract])) OR (Idiopathic Diffuse Interstitial Pulmonary Fibrosis[Title/Abstract]) \| 54,608 \| \| 3 \| "Pulmonary Fibrosis"[Mesh] \| 30,693 \| \| 2 \| ((((((Idiopathic Pulmonary Fibrosis[Title/Abstract]) OR (Idiopathic Pulmonary Fibroses[Title/Abstract])) OR (Pulmonary Fibroses, Idiopathic[Title/Abstract])) OR (Pulmonary Fibrosis, Idiopathic[Title/Abstract])) OR (Idiopathic Fibrosing Alveolitis, Chronic Form[Title/Abstract])) OR (Familial Idiopathic Pulmonary Fibrosis[Title/Abstract])) OR (Idiopathic Pulmonary Fibrosis, Familial[Title/Abstract]) \| 15,861 \| \| 1 \| "Idiopathic Pulmonary Fibrosis"[Mesh] \| 9,363 \| |
| --- | --- | --- | --- | --- | --- | --- | --- | --- | --- | --- | --- | --- | --- | --- | --- | --- | --- | --- | --- | --- | --- | --- | --- | --- | --- | --- | --- | --- | --- | --- | --- | --- | --- | --- | --- | --- | --- | --- | --- |
| **Embase**   \| No. \| Query \| Results \| Date \| \| --- \| --- \| --- \| --- \| \| #12 \| (('fibrosing alveolitis'/exp OR ('fibrosing alveolitis':ab,ti OR 'idiopathic pulmonary fibrosis':ab,ti OR 'idiopathic pulmonary fibroses':ab,ti OR 'pulmonary fibroses, idiopathic':ab,ti OR 'pulmonary fibrosis, idiopathic':ab,ti OR 'idiopathic fibrosing alveolitis, chronic form':ab,ti OR 'familial idiopathic pulmonary fibrosis':ab,ti OR 'idiopathic pulmonary fibrosis, familial':ab,ti) OR 'lung fibrosis'/exp OR ('lung fibrosis':ab,ti OR 'pulmonary fibrosis':ab,ti OR 'fibroses, pulmonary':ab,ti OR 'fibrosis, pulmonary':ab,ti OR 'pulmonary fibroses':ab,ti OR 'alveolitis, fibrosing':ab,ti OR 'alveolitides, fibrosing':ab,ti OR 'fibrosing alveolitides':ab,ti OR 'idiopathic diffuse interstitial pulmonary fibrosis':ab,ti)) AND ('drug therapy'/exp OR ('drug therapy':ab,ti OR 'pharmacotherapy':ab,ti OR 'pharmacotherapies':ab,ti OR 'therapy, drug':ab,ti OR 'drug therapies':ab,ti OR 'therapies, drug':ab,ti) OR ('pirfenidone':ab,ti OR 'nintedanib':ab,ti OR 'acetylcysteine':ab,ti OR 'colchicine':ab,ti OR 'endothelin receptor antagonist':ab,ti OR 'simtuzumab':ab,ti OR 'imatinib':ab,ti OR 'warfarin':ab,ti))) AND ('random':ab,ti OR 'placebo':ab,ti OR 'double-blind':ab,ti) \| 1356 \| 5-Jun-25 \| \| #11 \| 'random':ab,ti OR 'placebo':ab,ti OR 'double-blind':ab,ti \| 1040306 \| 5-Jun-25 \| \| #10 \| ('fibrosing alveolitis'/exp OR ('fibrosing alveolitis':ab,ti OR 'idiopathic pulmonary fibrosis':ab,ti OR 'idiopathic pulmonary fibroses':ab,ti OR 'pulmonary fibroses, idiopathic':ab,ti OR 'pulmonary fibrosis, idiopathic':ab,ti OR 'idiopathic fibrosing alveolitis, chronic form':ab,ti OR 'familial idiopathic pulmonary fibrosis':ab,ti OR 'idiopathic pulmonary fibrosis, familial':ab,ti) OR 'lung fibrosis'/exp OR ('lung fibrosis':ab,ti OR 'pulmonary fibrosis':ab,ti OR 'fibroses, pulmonary':ab,ti OR 'fibrosis, pulmonary':ab,ti OR 'pulmonary fibroses':ab,ti OR 'alveolitis, fibrosing':ab,ti OR 'alveolitides, fibrosing':ab,ti OR 'fibrosing alveolitides':ab,ti OR 'idiopathic diffuse interstitial pulmonary fibrosis':ab,ti)) AND ('drug therapy'/exp OR ('drug therapy':ab,ti OR 'pharmacotherapy':ab,ti OR 'pharmacotherapies':ab,ti OR 'therapy, drug':ab,ti OR 'drug therapies':ab,ti OR 'therapies, drug':ab,ti) OR ('pirfenidone':ab,ti OR 'nintedanib':ab,ti OR 'acetylcysteine':ab,ti OR 'colchicine':ab,ti OR 'endothelin receptor antagonist':ab,ti OR 'simtuzumab':ab,ti OR 'imatinib':ab,ti OR 'warfarin':ab,ti)) \| 20805 \| 5-Jun-25 \| \| #9 \| 'drug therapy'/exp OR ('drug therapy':ab,ti OR 'pharmacotherapy':ab,ti OR 'pharmacotherapies':ab,ti OR 'therapy, drug':ab,ti OR 'drug therapies':ab,ti OR 'therapies, drug':ab,ti) OR ('pirfenidone':ab,ti OR 'nintedanib':ab,ti OR 'acetylcysteine':ab,ti OR 'colchicine':ab,ti OR 'endothelin receptor antagonist':ab,ti OR 'simtuzumab':ab,ti OR 'imatinib':ab,ti OR 'warfarin':ab,ti) \| 4492663 \| 5-Jun-25 \| \| #8 \| 'pirfenidone':ab,ti OR 'nintedanib':ab,ti OR 'acetylcysteine':ab,ti OR 'colchicine':ab,ti OR 'endothelin receptor antagonist':ab,ti OR 'simtuzumab':ab,ti OR 'imatinib':ab,ti OR 'warfarin':ab,ti \| 133456 \| 5-Jun-25 \| \| #7 \| 'drug therapy':ab,ti OR 'pharmacotherapy':ab,ti OR 'pharmacotherapies':ab,ti OR 'therapy, drug':ab,ti OR 'drug therapies':ab,ti OR 'therapies, drug':ab,ti \| 139797 \| 5-Jun-25 \| \| #6 \| 'drug therapy'/exp \| 4330715 \| 5-Jun-25 \| \| #5 \| 'fibrosing alveolitis'/exp OR ('fibrosing alveolitis':ab,ti OR 'idiopathic pulmonary fibrosis':ab,ti OR 'idiopathic pulmonary fibroses':ab,ti OR 'pulmonary fibroses, idiopathic':ab,ti OR 'pulmonary fibrosis, idiopathic':ab,ti OR 'idiopathic fibrosing alveolitis, chronic form':ab,ti OR 'familial idiopathic pulmonary fibrosis':ab,ti OR 'idiopathic pulmonary fibrosis, familial':ab,ti) OR 'lung fibrosis'/exp OR ('lung fibrosis':ab,ti OR 'pulmonary fibrosis':ab,ti OR 'fibroses, pulmonary':ab,ti OR 'fibrosis, pulmonary':ab,ti OR 'pulmonary fibroses':ab,ti OR 'alveolitis, fibrosing':ab,ti OR 'alveolitides, fibrosing':ab,ti OR 'fibrosing alveolitides':ab,ti OR 'idiopathic diffuse interstitial pulmonary fibrosis':ab,ti) \| 117062 \| 5-Jun-25 \| \| #4 \| 'lung fibrosis':ab,ti OR 'pulmonary fibrosis':ab,ti OR 'fibroses, pulmonary':ab,ti OR 'fibrosis, pulmonary':ab,ti OR 'pulmonary fibroses':ab,ti OR 'alveolitis, fibrosing':ab,ti OR 'alveolitides, fibrosing':ab,ti OR 'fibrosing alveolitides':ab,ti OR 'idiopathic diffuse interstitial pulmonary fibrosis':ab,ti \| 51383 \| 5-Jun-25 \| \| #3 \| 'lung fibrosis'/exp \| 110547 \| 5-Jun-25 \| \| #2 \| 'fibrosing alveolitis':ab,ti OR 'idiopathic pulmonary fibrosis':ab,ti OR 'idiopathic pulmonary fibroses':ab,ti OR 'pulmonary fibroses, idiopathic':ab,ti OR 'pulmonary fibrosis, idiopathic':ab,ti OR 'idiopathic fibrosing alveolitis, chronic form':ab,ti OR 'familial idiopathic pulmonary fibrosis':ab,ti OR 'idiopathic pulmonary fibrosis, familial':ab,ti \| 25023 \| 5-Jun-25 \| \| #1 \| 'fibrosing alveolitis'/exp \| 39029 \| 5-Jun-25 \| |
| **Web of Science**   \| # \| Search Query \| Results \| \| --- \| --- \| --- \| \| 1 \| TS=(Idiopathic Pulmonary Fibrosis) OR TS=(Idiopathic Pulmonary Fibroses) OR TS=(Pulmonary Fibroses, Idiopathic) OR TS=(Pulmonary Fibrosis, Idiopathic) OR TS=(Idiopathic Fibrosing Alveolitis, Chronic Form) OR TS=(Familial Idiopathic Pulmonary Fibrosis) OR TS=(Idiopathic Pulmonary Fibrosis, Familial) \| 18289 \| \| 2 \| TS=(Pulmonary Fibrosis) OR TS=(Fibroses, Pulmonary) OR TS=(Fibrosis, Pulmonary) OR TS=(Pulmonary Fibroses) OR TS=(Alveolitis, Fibrosing) OR TS=(Alveolitides, Fibrosing) OR TS=(Fibrosing Alveolitides) OR TS=(Fibrosing Alveolitis) OR TS=(Idiopathic Diffuse Interstitial Pulmonary Fibrosis) \| 46101 \| \| 3 \| #1 OR #2 \| 46101 \| \| 4 \| TS=(Drug therapy) OR TS=(Pharmacotherapy) OR TS=(Pharmacotherapies) OR TS=(Therapy, Drug) OR TS=(Drug Therapies) OR TS=(Therapies, Drug) \| 398181 \| \| 5 \| TS=(pirfenidone) OR TS=(nintedanib) OR TS=(acetylcysteine) OR TS=(colchicine) OR TS=(endothelin receptor antagonist) OR TS=(simtuzumab) OR TS=(imatinib) OR TS=(warfarin) \| 73041 \| \| 6 \| #4 OR #5 \| 462553 \| \| 7 \| TS=(randomized controlled trial) OR TS=(randomized) OR TS=(placebo) OR TS=(random) OR TS=(double-blind) \| 1382553 \| \| 8 \| #3 AND #6 AND #7 \| 1184 \| |
| **Cochrane Library**  Date Run: 05/06/2025 09:34:35  Comment:  ID Search Hits  #1 MeSH descriptor: [Idiopathic Pulmonary Fibrosis] explode all trees 609  #2 (Idiopathic Pulmonary Fibrosis):ti,ab,kw OR (Idiopathic Pulmonary Fibroses):ti,ab,kw OR (Pulmonary Fibroses, Idiopathic):ti,ab,kw OR (Pulmonary Fibrosis, Idiopathic):ti,ab,kw OR (Idiopathic Fibrosing Alveolitis, Chronic Form):ti,ab,kw 1734  #3 (Familial Idiopathic Pulmonary Fibrosis):ti,ab,kw OR (Idiopathic Pulmonary Fibrosis, Familial):ti,ab,kw 10  #4 MeSH descriptor: [Pulmonary Fibrosis] explode all trees 932  #5 (Pulmonary Fibrosis):ti,ab,kw OR (Fibroses, Pulmonary):ti,ab,kw OR (Fibrosis, Pulmonary):ti,ab,kw OR (Pulmonary Fibroses):ti,ab,kw OR (Alveolitis, Fibrosing):ti,ab,kw 4735  #6 (Alveolitides, Fibrosing):ti,ab,kw OR (Fibrosing Alveolitides):ti,ab,kw OR (Fibrosing Alveolitis):ti,ab,kw OR (Idiopathic Diffuse Interstitial Pulmonary Fibrosis):ti,ab,kw 937  #7 #1 or #2 or #3 or #4 or #5 or #6 4746  #8 MeSH descriptor: [Drug Therapy] explode all trees 183271  #9 (Drug therapy):ti,ab,kw OR (Pharmacotherapy):ti,ab,kw OR (Pharmacotherapies):ti,ab,kw OR (Therapy, Drug):ti,ab,kw OR (Drug Therapies):ti,ab,kw 567476  #10 (Therapies, Drug):ti,ab,kw OR (pirfenidone):ti,ab,kw OR (nintedanib):ti,ab,kw OR (acetylcysteine):ti,ab,kw OR (colchicine):ti,ab,kw 29317  #11 (endothelin receptor antagonist):ti,ab,kw OR (simtuzumab):ti,ab,kw OR (imatinib):ti,ab,kw OR (Warfarin):ti,ab,kw 8075  #12 #8 or #9 or #10 or #11 619734  #13 #7 and #12 2379 |
| CNKI 595  检索式：  （主题：特发性肺间质纤维化 + 特发性肺纤维化 + 肺纤维化 + 肺间质纤维化）AND（主题：药物治疗 + 药物疗法 + 吡非尼酮 +尼达尼布 + N\-乙酰半胱氨酸 + 秋水仙碱 + 内质网受体拮抗剂 + LOXL2单抗 + 伊马替尼 + 华法林) |
| Wanfang 28  768  题名或关键词:(特发性肺间质纤维化 OR 特发性肺纤维化 OR 肺纤维化 OR 肺间质纤维化) and 题名或关键词:(药物治疗 OR 药物疗法 OR 吡非尼酮 OR 尼达尼布 OR N\-乙酰半胱氨酸 OR 秋水仙碱 OR 内质网受体拮抗剂 OR LOXL2单抗 OR 伊马替尼 OR 华法林) |
| VIP 629  ((((题名或关键词=特发性肺纤维化 OR 题名或关键词=特发性肺间质纤维化) OR 题名或关键词=肺纤维化) OR 题名或关键词=肺间质纤维化) AND (((((((((题名或关键词=药物治疗 OR 题名或关键词=药物疗法) OR 题名或关键词=吡非尼酮) OR 题名或关键词=尼达尼布) OR 题名或关键词=乙酰半胱氨酸) OR 题名或关键词=秋水仙碱) OR 题名或关键词=内质网受体拮抗剂) OR 题名或关键词=LOXL2单抗) OR 题名或关键词=伊马替尼) OR 题名或关键词=华法林)) |
| SinoMed 714  ( "特发性肺纤维化"[常用字段:智能] OR "特发性肺间质纤维化"[常用字段:智能] OR "肺纤维化"[常用字段:智能] OR "肺间质纤维化"[常用字段:智能]) AND( "药物治疗"[常用字段:智能] OR "药物疗法"[常用字段:智能] OR "吡非尼酮"[常用字段:智能] OR "尼达尼布)"[常用字段:智能] OR "乙酰半胱氨酸"[常用字段:智能] OR "秋水仙碱"[常用字段:智能] OR "内质网受体拮抗剂"[常用字段:智能] OR "LOXL2单抗"[常用字段:智能] OR "伊马替尼"[常用字段:智能] OR "华法林"[常用字段:智能])) |

# Supplementary Table S3 Data Characteristics Table

| First Author | Year | Country | Registration number | Patient source | Gender(I/C) | | | | Age(I/C) (years old) | | Sample size(I/C) | | Total sample size | Treatment(I/C) | | Dosage(I/C) | | Treatment time | Outcome | Number of adverse events(I/C) | |
| --- | --- | --- | --- | --- | --- | --- | --- | --- | --- | --- | --- | --- | --- | --- | --- | --- | --- | --- | --- | --- | --- |
|  |  |  |  |  | M(I) | F(I) | M(C) | F(C) |  |  |  |  |  |  |  |  |  |  |  |  |  |
| Zhanmin Zhang | 2005 | China |  | Sc | 14 | 6 | 10 | 4 | 65 | 65 | 20 | 14 | 34 | AZM | Placebo | 0.25g/d, p.o. qd | / | 3 months | FVC;VC;DLCO;FEV1/FVC | 3 | 6 |
| Maurits Demedts | 2005 | Belgium |  | Mc | 55 | 25 | 56 | 19 | 62±9 | 64±9 | 80 | 75 | 155 | NAC | Placebo | 1.8g/d, p.o. tid | / | 3 months | VC;DLCO | 72 | 67 |
| Lu Bai | 2006 | China |  | Sc | 16 | 8 | 12 | 12 | 49.5 | 51.5 | 24 | 24 | 48 | NAC | Placebo | 1.8g/d, p.o. tid | / | 3 months | VC;DLCO | / | / |
| Shaoxia Liu | 2006 | China |  | Sc | 10 | 8 | 8 | 6 | 63.9±9.4 | 65.6±8.7 | 18 | 14 | 32 | AZM | Placebo | 0.25g/d, p.o.qd | / | 2 months | FVC;DLCO | 3 | / |
| Dafang Wang | 2006 | China |  | Sc | 11 | 10 | 13 | 8 | 49-65 | 50-67 | 21 | 21 | 42 | BUD | Placebo | 3mg/d, inhal. tid | / | 3 months | VC | 0 | 11 |
| Zhihua Wang | 2006 | China |  | Sc | 17 | 6 | 18 | 6 | 57±8 | 59±8 | 23 | 24 | 47 | Captopril | Placebo | 75mg/d, p.o. tid | / | 12 months | VC;DLCO;TLC | / | / |
| Qianqian Nan | 2007 | China |  | Sc | 11 | 9 | 11 | 9 | 57±11 | 57±11 | 20 | 20 | 40 | NAC | Placebo | 1.8g/d, p.o. tid | / | 12 months | FVC;DLCO;FEV1/FVC | / | / |
| Zhigang Yang | 2008 | China |  | Sc | 11 | 6 | 9 | 6 | 61.3±10.2 | 61.3±10.2 | 17 | 15 | 32 | NAC | Placebo | 1.8g/d, p.o. tid | / | 12 months | FVC;DLCO;FEV1/FVC | 0 | 3 |
| Aigui Jiang | 2009 | China |  | Sc | 8 | 5 | 8 | 5 | 49.3±15.5 | 49.3±15.5 | 13 | 13 | 26 | NAC | Placebo | 1.8g/d, p.o. tid | / | 4/8/12 weeks | FVC;DLCO | / | / |
| Jianyong Zhu | 2009 | China |  | Sc | 6 | 5 | 6 | 5 | 65.7±4.8 | 65.7±4.8 | 10 | 11 | 21 | NAC | Placebo | 1.8g/d, p.o. tid | / | 6 months | VC;DLCO;TLC | 7 | 8 |
| Jianyong Zhu | 2009 | China |  | Sc | 6 | 5 | 6 | 5 | 65.7±4.8 | 65.7±4.8 | 11 | 11 | 22 | Captopril | Placebo | 25mg/d, p.o. tid | / | 6 months | VC;DLCO;TLC | 6 | 8 |
| Zhenjie Huang | 2010 | China |  | Sc | 14 | 8 | 12 | 8 | 62.1±11.2 | 62.1±11.2 | 22 | 20 | 42 | BUD+NAC | Placebo | BUD: 1mg/d, inhal. bid; NAC: 1.8g/d, p.o. tid | / | 4 weeks | FVC;DLCO;TLC;FEV1/FVC | / | / |
| Menghua Zuo | 2010 | China |  | Sc | 14 | 6 | 13 | 7 | 56.95±5.88 | 56.95±5.88 | 20 | 20 | 40 | NAC | Placebo | 1.8g/d, p.o. tid | / | 3 months | FVC;DLCO;FEV1/FVC | / | / |
| H. Taniguchi | 2010 | Japan |  | Mc | 85 | 23 | 81 | 23 | 65.4±6.2 | 64.7±7.3 | 108 | 104 | 212 | PFD | Placebo | 1.8g/d, p.o. tid | 0g/d, p.o. tid | 52 weeks | VC | 15 | 7 |
| H. Taniguchi | 2010 | Japan |  | Mc | 47 | 8 | 81 | 23 | 63.9±7.5 | 64.7±7.3 | 55 | 104 | 159 | PFD | Placebo | 1.2g/d, p.o. tid | 0g/d, p.o. tid | 52 weeks | VC | 9 | 7 |
| Qizhong Long | 2011 | China |  | Sc | 7 | 1 | 6 | 1 | 44.8±16.8 | 44.8±16.8 | 8 | 7 | 15 | NAC | Placebo | 1.8g/d, p.o. tid | / | 4/12/24 weeks | FVC;TLC;FEV1/FVC | / | / |
| Weilin Yang | 2011 | China |  | Sc | 22 | 16 | 13 | 7 | 72.3 | 74.1 | 38 | 20 | 58 | Thalidomide | Placebo | 50mg/d, p.o. qd | / | 24 weeks | FVC;DLCO;FEV1/FVC | / | / |
| Jing Zhu | 2011 | China |  | Sc | 13 | 7 | 12 | 8 | 54.5±3.1 | 49.3±3.7 | 20 | 20 | 40 | NAC | Placebo | 1.8g/d, p.o. tid | / | 12 months | VC;DLCO | / | / |
| Luca Richeldi^*^ | 2011 | Italy | NCT00514683 | Mc | 65 | 21 | 63 | 22 | 65.3±9.4 | 64.8±8.6 | 86 | 85 | 171 | Nintedanib | Placebo | 50mg/d, p.o.qd | / | 52 weeks | FVC | 26 | 26 |
| Luca Richeldi^*^ | 2011 | Italy | NCT00514683 | Mc | 62 | 24 | 63 | 22 | 64.9±8.5 | 64.8±8.6 | 86 | 85 | 171 | Nintedanib | Placebo | 50mg/d, p.o.bid | / | 52 weeks | FVC | 23 | 26 |
| Luca Richeldi^*^ | 2011 | Italy | NCT00514683 | Mc | 65 | 21 | 63 | 22 | 65.1±8.6 | 64.8±8.6 | 86 | 85 | 171 | Nintedanib | Placebo | 100mg/d, p.o. bid | / | 52 weeks | FVC | 18 | 26 |
| Luca Richeldi^*^ | 2011 | Italy | NCT00514683 | Mc | 65 | 20 | 63 | 22 | 65.4±7.8 | 64.8±8.6 | 85 | 85 | 170 | Nintedanib | Placebo | 200mg/d, p.o. bid | / | 52 weeks | FVC | 23 | 26 |
| Yanlei Ge | 2012 | China |  | Sc | 14 | 16 | 14 | 16 | 61.5±4.8 | 61.5±4.8 | 30 | 30 | 60 | IFN-γ1b | Placebo | 2,000,000units, s.c. qd | / | 2 months | VC;DLCO;TLC | / | / |
| Yingkun Sun | 2012 | China |  | Sc | 20 | 10 | 21 | 9 | 60.5±9.2 | 65.2±8.2 | 30 | 30 | 60 | NAC | Placebo | 0.4g/d, p.o. qd | / | 3 months | VC;TLC | / | / |
| Xuemei Yang | 2012 | China |  | Sc | 8 | 6 | 6 | 5 | 63±10.186 | 65±12.041 | 14 | 11 | 25 | MP | Placebo | 0-4week: 0.4mg/kg/d; 4-8week: 0.2mg/kg/d; 8week-: 0.1mg/kg/d | / | 12 months | FVC | 1 | 1 |
| Qing Yu | 2012 | China |  | Sc | 12 | 8 | 13 | 7 | 55.7±4.1 | 55.7±4.1 | 20 | 20 | 40 | CTX | Placebo | 150mg/d, p.o. | / | / | VC;DLCO;TLC | / | / |
| Qing Yu | 2012 | China |  | Sc | 12 | 8 | 13 | 7 | 55.7±4.1 | 55.7±4.1 | 20 | 20 | 40 | NAC | Placebo | 1.8g/d, p.o. tid | / | / | VC;DLCO;TLC | / | / |
| Rui Zhang | 2012 | China |  | Sc | / | / | / | / | / | / | 20 | 20 | 40 | NAC | Placebo | 1.8g/d, p.o. tid | / | 12 months | FVC;DLCO;FEV1/FVC | / | / |
| Sakae Homma^*^ | 2012 | Japan |  | Mc | 29 | 9 | 29 | 9 | 67.6±6.4 | 68.2±7.7 | 38 | 38 | 76 | NAC | Placebo | 704.8mg/d, inhal. bid | / | 12/24/36/48 weeks | FVC | 10 | 0 |
| Jianhua Lu | 2013 | China |  | Sc | / | / | / | / | 62.8±6.2 | 62.8±6.2 | 32 | 30 | 62 | NAC | Placebo | 1.8g/d, p.o. tid | / | 6 months | VC;DLCO;TLC | 2 | 0 |
| Keyun Shi | 2013 | China |  | Sc | 11 | 10 | 11 | 9 | 55.6±10.3 | 55.6±10.3 | 21 | 20 | 41 | NAC | Placebo | 0.6g/d, p.o. | / | 12 weeks | VC;DLCO;TLC;FEV1/FVC | / | / |
| Keyun Shi | 2013 | China |  | Sc | 11 | 10 | 11 | 9 | 55.6±10.3 | 55.6±10.3 | 21 | 20 | 41 | NAC+RXM | Placebo | NAC: 0.6g/d, p.o. ; RXM: 150mg/d, p.o. | / | 12 weeks | VC;DLCO;TLC;FEV1/FVC | / | / |
| Hua Li | 2014 | China |  | Sc | 6 | 7 | 5 | 8 | 54.6±9.4 | 54.6±9.4 | 13 | 13 | 26 | NAC | Placebo | 1.8g/d, p.o. tid | / | 1/2/3/4 weeks | FEV1/FVC | / | / |
| Xiuyan Li | 2014 | China |  | Sc | 29 | 19 | 29 | 19 | 61.3±7.8 | 61.3±7.8 | 48 | 48 | 96 | AZM | Placebo | 0.25g/d, p.o. qd | / | 3 months | FVC;DLCO | / | / |
| Haiying Zhang | 2014 | China |  | Sc | 34 | 21 | 33 | 22 | 36.5±5.3 | 36.9±5.5 | 55 | 55 | 110 | PDN | Placebo | 0.1mg/kg/d, p.o. | / | 1 year | FVC;DLCO;FEV1/FVC | 20 | 30 |
| Fernando J Martinez^*^ | 2014 | USA | NCT00650091 | Mc | 107 | 26 | 98 | 33 | 67.5±9.4 | 66.1±8.3 | 133 | 131 | 264 | NAC | Placebo | 1.8g/d, p.o. tid | / | 15/30/45/60 weeks | FVC | 25 | 20 |
| Luca Richeldi^*^ | 2014 | UK | NCT01335464 | Mc | 251 | 58 | 163 | 41 | 66.9±8.4 | 66.9±8.2 | 309 | 204 | 513 | Nintedanib | Placebo | 300mg/d, p.o. bid | / | 52 weeks | FVC | 96 | 55 |
| Luca Richeldi^*^ | 2014 | UK | NCT01335477 | Mc | 256 | 73 | 171 | 48 | 66.4±7.9 | 67.1±7.5 | 329 | 219 | 548 | Nintedanib | Placebo | 300mg/d, p.o. bid | / | 52 weeks | FVC | 98 | 72 |
| Songquan Fu | 2015 | China |  | Sc | 9 | 6 | 8 | 7 | 63.2±6.2 | 62.1±5.9 | 15 | 15 | 30 | NAC | Placebo | 0.6g/d, p.o. tid | / | 6 months | VC | / | / |
| Songquan Fu | 2015 | China |  | Sc | 8 | 7 | 8 | 7 | 64.1±6.5 | 62.1±5.9 | 15 | 15 | 30 | NAC | Placebo | 1.8g/d, p.o. tid | / | 6 months | VC | / | / |
| Qingyan Huang | 2015 | China |  | Sc | 19 | 18 | 20 | 17 | 62.3±12.8 | 61.7±13.5 | 37 | 37 | 74 | NAC | Placebo | 1.8g/d, p.o. tid | / | 6 months | FVC;DLCO;FEV1/FVC | 3 | 2 |
| Shanguo Jiang | 2015 | China |  | Sc | 7 | 8 | 8 | 7 | 63.8±6.1 | 64.3±7.1 | 15 | 15 | 30 | BUD+NAC | Placebo | BUD: 1mg/d, inhal. bid; NAC: 1.8g/d, p.o. tid | / | 4 weeks | TLC;FEV1/FVC | 0 | 0 |
| Wei Jin | 2015 | China |  | Sc | 13 | 12 | 15 | 10 | 65.45±5.21 | 65.44±5.21 | 25 | 25 | 50 | NAC | Placebo | 2-9ml/d, inhal. bid or tid | / | 1 month | FEV1/FVC | 0 | 0 |
| Huiping Li | 2015 | China |  | Mc | 36 | 7 | 39 | 5 | 61.9±6.0 | 62.6±6.9 | 43 | 44 | 87 | PFD | Placebo | 1.2g/d, p.o. tid | / | 12/24/36/48 weeks | FVC;DLCO | 36 | 32 |
| Yong Liu | 2015 | China |  | Sc | 31 | 9 | 33 | 7 | 54.2±12.5 | 51.4±10.8 | 40 | 40 | 80 | NAC | Placebo | 1.8g/d, p.o. tid | / | 3 months | FVC;DLCO;TLC;FEV1/FVC | 7 | 4 |
| Xianjun Ma | 2015 | China |  | Sc | 25 | 11 | 23 | 11 | 61.04±7.57 | 61.04±7.57 | 36 | 34 | 70 | NAC | Placebo | 1.8g/d, p.o. tid | / | 6 months | VC;DLCO;FEV1/FVC | / | / |
| Yan Shen | 2015 | China |  | Sc | 18 | 8 | 21 | 9 | 55.85±2.35 | 54.65±2.76 | 26 | 30 | 56 | AZM | Placebo | 0.25g/d, p.o. qd | / | 3/6 months | VC | 3 | 2 |
| Chuanhai Wang | 2015 | China |  | Sc | 13 | 8 | 11 | 9 | 55.8±2.6 | 55.3±3.9 | 21 | 21 | 42 | NAC | Placebo | 1.8g/d, p.o. tid | / | 6 months | VC;DLCO | 3 | 0 |
| Li Zhao | 2015 | China |  | Sc | 13 | 7 | 12 | 8 | 59.7±8.9 | 58.5±9.1 | 20 | 20 | 40 | NAC | Placebo | 1.8g/d, p.o. tid | / | 5 months | DLCO;TLC;FEV1/FVC | 7 | 13 |
| Hui Huang | 2015 | China | NCT01504334 | Mc | 33 | 5 | 38 | 0 | 59.03±5.94 | 61.61±5.94 | 38 | 38 | 76 | PFD | Placebo | 1.8g/d, p.o. tid | / | 24 weeks | FVC;DLCO;TLC | 2 | 2 |
| Yunxia Li | 2016 | China |  | Sc | 12 | 12 | 14 | 10 | 61.88±6.12 | 62.63±5.96 | 24 | 24 | 48 | PFD | Placebo | 1.2-1.8g/d, p.o. tid | / | 6 months | FVC;FEV1/FVC | 1 | 1 |
| Maohong Liu | 2016 | China |  | Sc | 19 | 11 | 17 | 13 | 60.2±7.6 | 60.4±7.3 | 30 | 30 | 60 | NAC | Placebo | 1.8g/d, p.o. tid | / | 6 months | FVC;VC | / | / |
| Xuejiao Liu | 2016 | China |  | Sc | 49 | 9 | 49 | 9 | 65.18±14.49 | 65.18±14.49 | 58 | 58 | 116 | NAC | Placebo | 1.8g/d, p.o. tid | / | 6 months | VC;DLCO;FEV1/FVC | / | / |
| Zheng Lu | 2016 | China |  | Sc | 8 | 10 | 7 | 11 | 64±12.4 | 63±11.12 | 18 | 18 | 36 | SASH | Placebo | 75mg/d, inhal. Tid | / | 1 month | FVC | 0 | 0 |
| Weihua Tian | 2016 | China |  | Sc | 26 | 25 | 27 | 24 | 45±5 | 44±6 | 51 | 51 | 102 | NAC | Placebo | 0.8-1.8g/d, p.o. bid/tid | / | 3 months | VC;TLC;FEV1/FVC | 3 | 4 |
| Chuanhai Wang | 2016 | China |  | Sc | 17 | 15 | 18 | 14 | 56.5±3.9 | 56.3±3.5 | 32 | 32 | 64 | NAC+MK0476 | Placebo | NAC: 1.8g/d, p.o. tid;MK0476: 10mg/d, p.o qd | / | 3 months | VC;DLCO | 4 | / |
| Feng Xu | 2016 | China |  | Sc | 14 | 16 | 16 | 14 | 56.2±2.7 | 59.5±2.5 | 30 | 30 | 60 | NAC+MK0476 | Placebo | NAC: 1.8g/d, p.o. tid;MK0476: 10mg/d, p.o qd | / | 6 months | FVC;DLCO | 2 | 0 |
| Jürgen Behr^*^ | 2016 | Germany | EudraCT number 2012-000564-14 | Mc | 53 | 7 | 51 | 11 | 66.7±8.0 | 67.5±6.2 | 60 | 62 | 122 | NAC | Placebo | 1.8g/d, p.o. tid | / | 24 weeks | FVC;DLCO | 46 | 50 |
| Hongmei Chen | 2017 | China |  | Sc | 15 | 17 | 16 | 16 | 45.18±6.34 | 46.07±6.59 | 32 | 32 | 64 | NAC | Placebo | 1.8g/d, p.o. tid | / | 2 weeks | VC;TLC;FEV1/FVC | 2 | 8 |
| Suping Guo | 2017 | China |  | Sc | 27 | 22 | 29 | 20 | 51.83±7.27 | 52.94±8.15 | 49 | 49 | 98 | PFD | Placebo | 1.2-1.8g/d, p.o. tid | / | 3 months | FVC | / | / |
| Qingshuang Mu | 2017 | China |  | Sc | 8 | 7 | 9 | 6 | 61.7±6.4 | 62.3±7.2 | 15 | 15 | 30 | NAC | Placebo | 1.8g/d, p.o. tid | / | 8 weeks | TLC;FEV1/FVC | 0 | 6 |
| Junxia Wang | 2017 | China |  | Sc | 17 | 18 | 18 | 17 | 54.89±2.13 | 55.27±2.88 | 35 | 35 | 70 | NAC+MK0476 | Placebo | NAC: 1.8g/d, p.o. tid;MK0476: 10mg/d, p.o qd | / | 3 months | FVC;DLCO | / | / |
| Jing Xu | 2017 | China |  | Sc | 15 | 15 | 16 | 14 | 63.4±7.1 | 62.3±6.8 | 30 | 30 | 60 | NAC | Placebo | 0.2g/d, p.o. qd | / | 4 months | FVC | / | / |
| Jing Xu | 2017 | China |  | Sc | 17 | 13 | 16 | 14 | 61.8±6.2 | 62.3±6.8 | 30 | 30 | 60 | NAC | Placebo | 0.6g/d, p.o. qd | / | 4 months | FVC | / | / |
| Arata Azuma | 2017 | Japan | NCT01335464 | Mc | 62 | 14 | 40 | 10 | 68.4±7.6 | 68.2±6.2 | 76 | 50 | 126 | Nintedanib | Placebo | 300mg/d, p.o. bid | / | 52 weeks | FVC | 14 | 9 |
| Arata Azuma | 2017 | Japan | NCT01335477 | Mc | 507 | 131 | 334 | 89 | 66.6±8.1 | 67.0±7.9 | 638 | 423 | 1061 | Nintedanib | Placebo | 300mg/d, p.o. bid | / | 52 weeks | FVC | 174 | 99 |
| Ganesh Raghu^*^ | 2017 | USA | NCT01769196 | Mc | 227 | 45 | 225 | 47 | 67.7±7.6 | 68.5±7.1 | 272 | 272 | 544 | Simtuzumab | Placebo | 1 ml/w, s.c. qw | / | 24 weeks | FVC | 31 | 32 |
| Yusheng Chai | 2018 | China |  | Sc | 48 | 42 | 47 | 43 | 54.69±4.92 | 55.37±5.18 | 90 | 90 | 180 | MK0476 | Placebo | MK0476: 10mg/d, p.o qd | / | 3 months | FVC | / | / |
| Kaichun Lei | 2018 | China |  | Sc | 17 | 3 | 13 | 7 | 64.6±10.4 | 71.4±10.2 | 20 | 20 | 40 | PFD | Placebo | 1.2g/d, p.o. tid | / | 12/24/36/48 weeks | FVC;DLCO | 13 | 0 |
| Huili Li | 2018 | China |  | Sc | 19 | 10 | 20 | 9 | 60.67±10.83 | 61.03±11.04 | 29 | 29 | 58 | NAC | Placebo | 1.8g/d, p.o. tid | / | 3 months | FVC;FEV1/FVC | / | / |
| Jingpan Li | 2018 | China |  | Sc | 17 | 9 | 19 | 7 | 67±3.4 | 67±3.4 | 26 | 26 | 52 | NAC | Placebo | 1.8g/d, p.o. tid | / | 3 months | VC | 0 | 1 |
| Zhenxing Mao | 2018 | China |  | Sc | 22 | 13 | 21 | 11 | 59.84±13.37 | 58.18±8.97 | 35 | 32 | 67 | PFD | Placebo | 1.8g/d, p.o. tid | / | 4 months | FVC | 12 | 7 |
| Fanqiu Meng | 2018 | China |  | Sc | 23 | 17 | 21 | 19 | 37.0±8.2 | 35.0±6.2 | 30 | 30 | 60 | NAC | Placebo | 0.6g/d, p.o. qd | / | 2 months | FVC | / | / |
| Guangchao Shi | 2018 | China |  | Sc | 20 | 18 | 18 | 19 | 53.6±4.2 | 53.7±3.9 | 38 | 37 | 75 | PFD | Placebo | 600mg/d, p.o. tid | / | 4 months | FVC;FEV1/FVC | / | / |
| Martin Kolb | 2018 | Canada |  | Mc | 110 | 27 | 106 | 20 | 70.3±8.6 | 70.0±7.9 | 137 | 136 | 273 | Sildenafil | Placebo | 60mg/d, p.o. tid | / | 12/24 weeks | FVC;DLCO | 133 | 127 |
| Luca Richeldi^*^ | 2018 | UK | NCT00514683 | Mc | 65 | 21 | 63 | 22 | 65.3±9.42 | 64.8±8.57 | 86 | 85 | 171 | Nintedanib | Placebo | 50mg/d, p.o.qd | / | 52 weeks | FVC | 26 | 26 |
| Luca Richeldi^*^ | 2018 | UK | NCT00514683 | Mc | 62 | 24 | 63 | 22 | 64.9±8.48 | 64.8±8.57 | 86 | 85 | 171 | Nintedanib | Placebo | 100mg/d, p.o. bid | / | 52 weeks | FVC | 23 | 26 |
| Luca Richeldi^*^ | 2018 | UK | NCT00514683 | Mc | 65 | 21 | 63 | 22 | 65.1±8.63 | 64.8±8.57 | 86 | 85 | 171 | Nintedanib | Placebo | 200mg/d, p.o. bid | / | 52 weeks | FVC | 18 | 26 |
| Luca Richeldi^*^ | 2018 | UK | NCT00514683 | Mc | 65 | 20 | 63 | 22 | 65.4±7.82 | 64.8±8.57 | 86 | 85 | 171 | Nintedanib | Placebo | 300mg/d, p.o. bid | / | 52 weeks | FVC | 23 | 26 |
| Luca Richeldi^*^ | 2018 | UK | NCT01170065 | Sc | 90 | 36 | 23 | 14 | 65.4±8.6 | 64.2±7.3 | 126 | 37 | 163 | Nintedanib | Placebo | 50-200mg/d, p.o. qd-bid | / | 61.8 months | FVC | 94 | 25 |
| Luca Richeldi^*^ | 2018 | UK | NCT01170065 | Sc | 28 | 7 | 23 | 14 | 65.2±7.2 | 64.2±7.3 | 35 | 37 | 72 | Nintedanib | Placebo | 300mg/d, p.o. bid | / | 61.8 months | FVC | 22 | 25 |
| Carlo Vancheri | 2018 | Italy | NCT02579603 | Sc | 42 | 11 | 44 | 7 | 68.9±6.6 | 68.9±6.8 | 53 | 51 | 104 | PFD | Placebo | 801mg/d, p.o. tid | / | 12 weeks | FVC | 47 | 45 |
| Lu Cao | 2018 | China |  | Sc | 19 | 21 | 16 | 24 | 54.06 | 54.12 | 40 | 40 | 80 | NAC | Placebo | 1.8g/d, p.o. tid | / | 2 weeks | FVC | 5 | 4 |
| Shimou Chen | 2019 | China |  | Sc | 30 | 21 | 28 | 23 | 47.2±3.1 | 45.3±6.5 | 51 | 51 | 102 | NAC | Placebo | 0.4-0.6g/d, p.o. bid/tid | / | 3 months | VC;TLC;FEV1/FVC | 3 | 6 |
| Junxing Zhu | 2019 | China |  | Sc | 11 | 14 | 15 | 10 | 60.58±5.66 | 60.55±5.89 | 25 | 25 | 50 | BUD | Placebo | 0.6mg/d, inhal. Bid | / | 3 months | FVC | 2 | 7 |
| Huajun Guo | 2019 | China |  | Sc | 24 | 17 | 25 | 16 | 59.57±11.33 | 59.44±12.45 | 41 | 41 | 82 | PFD | Placebo | 1800mg/d, p.o. tid | / | 4 months | FVC | / | / |
| Chunlian Liang | 2019 | China |  | Sc | 22 | 8 | 20 | 10 | 63.0±5.7 | 65.4±4.9 | 30 | 30 | 60 | PFD | Placebo | 1200mg/d, p.o. tid | / | 6 months | FVC;DLCO | 11 | 0 |
| Fenli Wang | 2019 | China |  | Sc | 30 | 25 | 31 | 24 | 57.92±11.81 | 59.83±12.54 | 55 | 55 | 110 | PFD | Placebo | 1.2g/d, p.o. tid | / | 36 weeks | FVC;DLCO | 5 | 2 |
| Jianying Wen | 2019 | China |  | Sc | 23 | 20 | 24 | 19 | 56.24±10.24 | 55.63±10.54 | 43 | 43 | 86 | NAC | Placebo | 1.8g/d, p.o. tid | / | 6 months | FVC;DLCO | 0 | 0 |
| Baomin Wu | 2019 | China |  | Sc | 19 | 17 | 20 | 16 | 57.3±4.8 | 56.8±5.2 | 36 | 36 | 72 | NAC+MK0476 | Placebo | NAC: 1.8g/d, p.o. tid;MK0476: 10mg/d, p.o qd | / | 6 months | VC;DLCO | / | / |
| Xiaojuan Yang | 2019 | China |  | Sc | 14 | 25 | 21 | 18 | 57.32±14.56 | 57.28±14.03 | 39 | 39 | 78 | NAC | Placebo | 1.8g/d, p.o. tid | / | 3 months | FVC;VC;TLC | / | / |
| Chengjie Zhao | 2019 | China |  | Sc | 26 | 10 | 25 | 11 | 53.51±3.76 | 52.67±3.12 | 36 | 36 | 72 | NAC+PFD | Placebo | NAC+PFD:1.8g/d, p.o. tid | / | 6 months | FVC | 0 | 0 |
| Liping Liang | 2020 | China |  | Sc | 17 | 13 | 12 | 10 | 71.6±10.1 | 72.0±9.8 | 30 | 22 | 52 | PFD | Placebo | 1800mg/d, p.o. tid | / | 3 months | FVC;DLCO;TLC | / | / |
| Feng Lin | 2020 | China |  | Sc | 20 | 20 | 21 | 19 | 57.56±5.24 | 57.85±5.42 | 40 | 40 | 80 | PFD | Placebo | 1200-1800mg/d, p.o. tid | / | 16 weeks | FVC;FEV1/FVC | / | / |
| Fenling Zhang | 2020 | China |  | Sc | 24 | 24 | 25 | 22 | 61.00±5.10 | 61.01±5.05 | 48 | 47 | 95 | NAC | Placebo | 1.2g/d, p.o. tid | / | 10 days | DLCO;FEV1/FVC | / | / |
| Zhenzhen Zhang | 2020 | China |  | Sc | 18 | 12 | 16 | 14 | 60.1±2.4 | 61.3±1.4 | 30 | 30 | 60 | PFD | Placebo | 600mg/d, p.o. tid | / | 16 weeks | FVC | 3 | 2 |
| Yuxian Zhao | 2020 | China |  | Sc | 21 | 19 | 22 | 18 | 45.1±1.33 | 45.02±1.32 | 40 | 40 | 80 | NAC | Placebo | 1.8g/d, p.o. tid | / | 3 months | FVC | / | / |
| Yongyi Zou | 2020 | China |  | Sc | 28 | 13 | 27 | 14 | 61.35±6.87 | 61.58±6.79 | 41 | 41 | 82 | PFD | Placebo | 600mg/d, p.o. tid | / | 3 months | FVC | / | / |
| Lisa Lancaster | 2020 | USA |  | Sc | 45 | 11 | 37 | 20 | 68.8±7.6 | 66.2±9.4 | 56 | 57 | 113 | Nintedanib | Placebo | 300mg/d, p.o. bid | / | 6 months | FVC | 55 | 51 |
| Luca Richeldi^*^ | 2020 | Italy | NCT01335464;NCT01335477 | Mc | 507 | 131 | 334 | 89 | 66.6±8.1 | 67.0±7.9 | 638 | 423 | 1061 | Nintedanib | Placebo | 300mg/d, p.o. bid | / | 24 weeks | FVC | 580 | 345 |
| Luca Richeldi^*^ | 2020 | Italy | NCT01890265 | Mc | 33 | 17 | 43 | 10 | 68.3±7.05 | 68.4±7.20 | 50 | 53 | 103 | Pamrevlumab | Placebo | 30mg/kg, i.v.gtt. Q3w | / | 12/24/36/48 weeks | FVC | 48 | 52 |
| Jin Woo Song | 2020 | South Korea | NCT01335464;NCT01335477 | Mc | 154 | 40 | 105 | 23 | 66.0±8.1 | 66.0±7.1 | 194 | 128 | 322 | Nintedanib | Placebo | 300mg/d, p.o. bid | / | 52 weeks | FVC | 181 | 110 |
| Zhenzhen Tao | 2021 | China |  | Sc | 24 | 18 | 22 | 20 | 48.63±8.01 | 47.81±7.52 | 42 | 42 | 84 | PFD | Placebo | 1800mg/d, p.o. tid | / | 4 months | FVC | / | / |
| Lina Wang | 2021 | China |  | Sc | 29 | 21 | 28 | 22 | 52.8±6.1 | 52.3±6.5 | 50 | 50 | 100 | Thalidomide | Placebo | 0-3 month: 100mg/d, p.o. qd; 3-6 month:mg/d, p.o. qd | / | 6 months | FVC;DLCO | / | / |
| Sue Wang | 2021 | China |  | Sc | 24 | 19 | 23 | 19 | 58.21±3.47 | 58.17±3.52 | 43 | 42 | 85 | NAC | Placebo | 1.8g/d, p.o. tid | / | 6 months | DLCO | / | / |
| Xiaodan Zhou | 2021 | China |  | Sc | 34 | 4 | 35 | 5 | 65.81±8.76 | 66.45±9.23 | 38 | 40 | 78 | NAC | Placebo | 1.8g/d, p.o. tid | / | 6 months | FVC;DLCO | 10 | 12 |
| Guanda Chen | 2022 | China |  | Sc | 23 | 21 | 24 | 20 | 55.79±10.37 | 55.86±10.24 | 44 | 44 | 88 | PFD | Placebo | 1800mg/d, p.o. tid | / | 4 months | FVC | / | / |
| Ze Deng | 2022 | China |  | Sc | 27 | 17 | 24 | 20 | 51.19±10.37 | 50.34±6.78 | 44 | 44 | 88 | Nintedanib | Placebo | 300mg/d, p.o. bid | / | 12 weeks | FVC;DLCO | 9 | 7 |
| Zhong Guo | 2022 | China |  | Sc | 31 | 33 | 30 | 34 | 47.28±2.36 | 46.28±2.15 | 64 | 64 | 128 | NAC | Placebo | 0.4-0.6g/d, p.o. bid/tid |  | 12 weeks | VC;TLC;FEV1/FVC | 3 | 4 |
| Fumei He | 2022 | China |  | Sc | 13 | 11 | 14 | 10 | 52.25±10.41 | 51.37±9.41 | 24 | 24 | 48 | NAC+PFD | Placebo | NAC+PFD:1.8g/d, p.o. tid | / | 6 months | DLCO | / | / |
| Lu Song | 2022 | China |  | Sc | 28 | 22 | 26 | 24 | 51.02±8.54 | 51.69±8.31 | 50 | 50 | 100 | Nintedanib | Placebo | 300mg/d, p.o. bid | / | 12 weeks | FEV1/FVC | 2 | 1 |
| Zehai Xia | 2022 | China |  | Sc | 19 | 1 | 18 | 2 | 65.41±8.24 | 68.07±7.36 | 20 | 20 | 40 | PFD | Placebo | 1200-1800mg/d, p.o. | / | 6/12 months | FVC;DLCO | 9 | 0 |
| Aijing Yang | 2022 | China |  | Sc | 15 | 9 | 13 | 11 | 61.24±2.03 | 61.52±1.97 | 24 | 24 | 48 | PFD | Placebo | 600mg/d, p.o. tid | / | 2 months | FVC | / | / |
| Weiwei Chen | 2023 | China |  | Sc | 25 | 16 | 27 | 14 | 60.25±11.43 | 60.23±11.39 | 41 | 41 | 82 | PFD | Placebo | 1800mg/d, p.o. tid | / | 6 months | FVC | 4 | 2 |
| Haitao Hu | 2023 | China |  | Sc | 30 | 16 | 28 | 18 | 64.98±5.38 | 65.28±5.43 | 46 | 46 | 92 | PFD | Placebo | 1800mg/d, p.o. tid | / | 12 weeks | FVC;DLCO | 7 | 5 |
| Shuai Wang | 2023 | China |  | Sc | 67 | 25 | 36 | 16 | 67.4±6.8 | 68.2±5.2 | 92 | 52 | 144 | Nintedanib | Placebo | 300mg/d, p.o. bid | / | 24 weeks | FVC;DLCO | 20 | 12 |
| Yanhua Wang | 2023 | China |  | Sc | 12 | 8 | 11 | 9 | 65.07±4.33 | 65.01±4.37 | 20 | 20 | 40 | BUD+NAC | Placebo | BUD: 1mg/d, inhal. bid; NAC: 1.2g/d, p.o. bid | / | 4 weeks | FVC;DLCO;TLC;FEV1/FVC | / | / |
| Chanchan Xu | 2023 | China |  | Sc | 25 | 12 | 21 | 15 | 58.39±7.14 | 57.93±7.20 | 37 | 36 | 73 | NAC | Placebo | 0.6-1.2g/d, p.o. qd/bid | / | 12 weeks | FVC;FEV1/FVC | 0 | 0 |
| Hongmei Zhao | 2023 | China |  | Sc | 14 | 7 | 15 | 6 | 64.3±9.2 | 61.6±8.8 | 21 | 21 | 42 | NAC | Placebo | 6ml/d, inhal. bid | / | 24 weeks | FVC;DLCO | 13 | 11 |
| Haiyan Chen | 2024 | China |  | Sc | 26 | 14 | 27 | 13 | 64.27±3.45 | 64.73±3.19 | 40 | 40 | 80 | Ambroxol | Placebo | 90mg/d, p.o. tid | / | 3 months | DLCO;TLC | 0 | 0 |
| Jia Chen | 2024 | China |  | Sc | 22 | 23 | 23 | 22 | 60.92±5.79 | 60.85±5.82 | 45 | 45 | 90 | NAC | Placebo | 1.8g/d, p.o. tid | / | 3 months | FVC;DLCO;TLC;FEV1/FVC | / | / |
| Wei Kong | 2024 | China |  | Sc | 28 | 21 | 26 | 23 | 63.1±15.6 | 62.6±15.5 | 49 | 49 | 98 | Nintedanib | Placebo | 300mg/d, p.o. bid | / | 3 months | FVC;FEV1/FVC | / | / |
| Ying Lei | 2024 | China |  | Sc | 15 | 19 | 23 | 11 | 49.67±3.21 | 49.86±3.17 | 34 | 34 | 68 | NAC | Placebo | 0.6-1.2g/d, p.o. qd/bid | / | 3 months | FVC;FEV1/FVC | / | / |
| Zhenxiang Liu | 2024 | China |  | Sc | 28 | 22 | 29 | 21 | 56.45±8.42 | 56.73±8.33 | 50 | 50 | 100 | NAC | Placebo | 1.8g/d, p.o. tid | / | 6 months | FVC | 2 | 10 |
| Yongjie Qie | 2024 | China |  | Sc | 28 | 15 | 23 | 14 | 61.64±6.75 | 62.38±7.03 | 43 | 37 | 80 | PFD | Placebo | 600mg/d, p.o. tid | / | 12 weeks | VC;TLC | / | / |
| Huanqin Wang | 2024 | China |  | Sc | 37 | 18 | 35 | 20 | 50.02±2.65 | 50.38±2.47 | 55 | 55 | 110 | PFD | Placebo | 1800mg/d, p.o. tid | / | 3 months | FEV1/FVC | 7 | 5 |
| Xizhi Wang | 2024 | China |  | Sc | 24 | 19 | 26 | 17 | 62.59±4.76 | 62.48±4.72 | 43 | 43 | 86 | NAC | Placebo | 1.8g/d, p.o. tid | / | 3 months | FVC;DLCO | 9 | 6 |
| Zhuqiang Wu | 2024 | China |  | Sc | 23 | 20 | 22 | 21 | 48.27±2.35 | 49.37±2.46 | 43 | 43 | 86 | Nintedanib | Placebo | 200mg/d, p.o. bid | / | 3 months | VC;TLC | / | / |
| Lifei Yue | 2024 | China |  | Sc | 24 | 16 | 23 | 17 | 54.34±6.24 | 53.82±5.75 | 30 | 30 | 60 | PFD | Placebo | 600mg/d, p.o. tid | / | 60 days | FVC | 8 | 6 |
| Yanni Zhang | 2024 | China |  | Sc | 23 | 13 | 20 | 16 | 56.05±3.58 | 55.94±3.64 | 36 | 36 | 72 | Metformin | Placebo | 500mg/d, p.o. qd | / | 12 months | FVC | 4 | 3 |
| Jing Zhao | 2024 | China |  | Sc | 33 | 19 | 30 | 22 | 68.62±4.39 | 69.08±4.23 | 52 | 52 | 104 | Thalidomide | Placebo | 100mg/d, p.o. bid | / | 3 months | FVC;FEV1/FVC | 5 | 8 |
| Xuan Zhou | 2024 | China |  | Sc | 57 | 30 | 44 | 21 | 52.76±8.91 | 52.76±8.91 | 87 | 65 | 152 | Nintedanib | PFD | 300mg/d, p.o. bid | 1800mg/d, p.o. tid | 6 months | FVC;DlCO | / | / |
| Zhengxue Zhu | 2024 | China |  | Sc | 26 | 14 | 25 | 15 | 63.13±0.69 | 63.29±0.72 | 40 | 40 | 80 | Nintedanib | Placebo | 300mg/d, p.o. bid | / | 3 months | FVC | / | / |
| Zulipeiya | 2024 | China |  | Sc | 22 | 12 | 18 | 15 | 61.20±9.89 | 59.04±10.11 | 34 | 33 | 67 | NAC | Placebo | 0.6g/d, p.o. tid | / | 4 months | FVC;DLCO | / | / |
| Ganesh Raghu | 2024 | USA | NCT03955146 | Mc | 132 | 49 | 126 | 49 | 67.3 | 69 | 181 | 175 | 356 | Pamrevlumab | Placebo | 30mg/kg, i.v.gtt. Q3w | / | 48 weeks | FVC | 23 | 22 |
| Cuijing Zhang | 2025 | China |  | Sc | 20 | 12 | 20 | 12 | 66.6±3.2 | 65.8±3.1 | 32 | 32 | 64 | PFD | Placebo | 600mg/d, p.o. tid | / | 8 weeks | VC | / | / |
| Toby M Maher^*^ | 2025 | USA | NCT05321082 | Mc | 220 | 171 | 231 | 161 | 66.0±9.8 | 66.6±10.3 | 391 | 392 | 783 | Nerandomilast | Placebo | 36mg/d, p.o. bid | / | 52 weeks | FVC | / | / |
| Toby M Maher^*^  *: outcome datas were presented exclusively in graphical form | 2025 | USA | NCT05321082 | Mc | 203 | 190 | 231 | 161 | 66.5±9.8 | 66.6±10.3 | 393 | 392 | 785 | Nerandomilast | Placebo | 18mg/d, p.o. bid | / | 52 weeks | FVC | / | / |

# Supplementary Table S4 league tables

### **Supplementary Table S4A league table of FVC**

| AZM(36.68%) |  |  |  |  |  |  |  |  |  |  |  |  |  |  |  |  |  |  |
| --- | --- | --- | --- | --- | --- | --- | --- | --- | --- | --- | --- | --- | --- | --- | --- | --- | --- | --- |
| -1.06 (-4.53, 2.39) | BUD(57.96%) |  |  |  |  |  |  |  |  |  |  |  |  |  |  |  |  |  |
| -0.03 (-2.71, 2.64) | 1.04 (-2.75, 4.82) | BUD_NAC(37.95%) |  |  |  |  |  |  |  |  |  |  |  |  |  |  |  |  |
| -1.84 (-5.29, 1.61) | -0.77 (-5.15, 3.59) | -1.82 (-5.58, 1.98) | Metformin(70.91%) |  |  |  |  |  |  |  |  |  |  |  |  |  |  |  |
| 1.82 (-1.57, 5.25) | 2.88 (-1.43, 7.22) | 1.84 (-1.88, 5.6) | 3.65 (-0.65, 7.99) | MK0476(11.68%) |  |  |  |  |  |  |  |  |  |  |  |  |  |  |
| 0.74 (-2.75, 4.23) | 1.8 (-2.58, 6.22) | 0.77 (-3.02, 4.6) | 2.59 (-1.81, 6.97) | -1.08 (-5.44, 3.28) | MP(26.15%) |  |  |  |  |  |  |  |  |  |  |  |  |  |
| 0.09 (-1.53, 1.72) | 1.16 (-1.99, 4.31) | 0.12 (-2.12, 2.35) | 1.93 (-1.2, 5.05) | -1.73 (-4.82, 1.34) | -0.65 (-3.83, 2.52) | NAC(33.77%) |  |  |  |  |  |  |  |  |  |  |  |  |
| 0.35 (-2.3, 3.02) | 1.42 (-2.37, 5.21) | 0.38 (-2.68, 3.45) | 2.2 (-1.58, 5.93) | -1.47 (-5.2, 2.26) | -0.39 (-4.19, 3.41) | 0.26 (-1.96, 2.49) | NAC_MK0476(30.14%) |  |  |  |  |  |  |  |  |  |  |  |
| -1.11 (-4.55, 2.31) | -0.04 (-4.39, 4.31) | -1.07 (-4.85, 2.67) | 0.73 (-3.63, 5.07) | -2.93 (-7.27, 1.37) | -1.86 (-6.24, 2.52) | -1.2 (-4.33, 1.92) | -1.46 (-5.24, 2.3) | NAC_PFD(58.81%) |  |  |  |  |  |  |  |  |  |  |
| **-4.83 (-7.47, -2.19)** | -3.77 (-7.53, 0) | **-4.8 (-7.86, -1.74)** | -2.99 (-6.75, 0.76) | **-6.65 (-10.37, -2.93)** | **-5.57 (-9.37, -1.78)** | **-4.92 (-7.12, -2.72)** | **-5.18 (-8.23, -2.14)** | -3.72 (-7.46, 0.04) | Nerandomilast(98.85%) |  |  |  |  |  |  |  |  |  |
| **-2.02 (-3.7, -0.35)** | -0.96 (-4.13, 2.21) | -2 (-4.27, 0.27) | -0.18 (-3.34, 2.96) | **-3.84 (-6.96, -0.74)** | -2.77 (-5.97, 0.42) | **-2.12 (-2.94, -1.29)** | **-2.38 (-4.65, -0.11)** | -0.92 (-4.06, 2.24) | **2.81 (0.56, 5.05)** | Nintedanib(80.39%) |  |  |  |  |  |  |  |  |
| -1.59 (-3.67, 0.46) | -0.53 (-3.92, 2.84) | -1.57 (-4.14, 1.01) | 0.24 (-3.13, 3.61) | -3.42 (-6.76, -0.1) | -2.34 (-5.77, 1.06) | -1.69 (-3.15, -0.23) | -1.95 (-4.51, 0.61) | -0.49 (-3.85, 2.89) | **3.23 (0.69, 5.77)** | 0.42 (-1.09, 1.95) | Pamrevlumab(71.73%) |  |  |  |  |  |  |  |
| -0.16 (-3.58, 3.25) | 0.9 (-3.42, 5.22) | -0.14 (-3.89, 3.6) | 1.67 (-2.67, 6) | -1.99 (-6.29, 2.31) | -0.91 (-5.25, 3.44) | -0.25 (-3.35, 2.83) | -0.52 (-4.27, 3.21) | 0.95 (-3.38, 5.29) | **4.67 (0.93, 8.39)** | 1.86 (-1.26, 4.98) | 1.44 (-1.91, 4.78) | PDN(41.59%) |  |  |  |  |  |  |
| -0.19 (-1.84, 1.45) | 0.87 (-2.28, 4.02) | -0.16 (-2.42, 2.09) | 1.64 (-1.49, 4.77) | -2.01 (-5.12, 1.07) | -0.93 (-4.13, 2.24) | -0.28 (-1.04, 0.47) | -0.54 (-2.79, 1.69) | 0.91 (-2.21, 4.05) | **4.64 (2.42, 6.85)** | **1.83 (0.99, 2.68)** | 1.41 (-0.07, 2.89) | -0.03 (-3.13, 3.07) | PFD(41.88%) |  |  |  |  |  |
| 0.71 (-0.83, 2.26) | 1.78 (-1.32, 4.88) | 0.74 (-1.45, 2.91) | 2.55 (-0.54, 5.63) | -1.11 (-4.15, 1.92) | -0.03 (-3.17, 3.1) | **0.62 (0.12, 1.13)** | 0.36 (-1.81, 2.52) | 1.82 (-1.25, 4.91) | **5.54 (3.4, 7.68)** | **2.74 (2.08, 3.39)** | **2.31 (0.95, 3.68)** | 0.88 (-2.17, 3.93) | **0.91 (0.34, 1.47)** | Placebo(17.30%) |  |  |  |  |
| -1.36 (-4.87, 2.12) | -0.3 (-4.72, 4.08) | -1.33 (-5.16, 2.49) | 0.48 (-3.93, 4.88) | -3.18 (-7.58, 1.16) | -2.11 (-6.51, 2.31) | -1.45 (-4.63, 1.71) | -1.71 (-5.52, 2.1) | -0.25 (-4.63, 4.15) | 3.47 (-0.34, 7.26) | 0.66 (-2.56, 3.85) | 0.24 (-3.19, 3.64) | -1.19 (-5.58, 3.19) | -1.17 (-4.36, 2.01) | -2.07 (-5.22, 1.06) | SASH(63.07%) |  |  |  |
| -1.75 (-4.4, 0.91) | -0.69 (-4.45, 3.08) | -1.72 (-4.79, 1.34) | 0.09 (-3.66, 3.84) | -3.57 (-7.31, 0.15) | -2.5 (-6.28, 1.29) | -1.84 (-4.04, 0.37) | -2.11 (-5.15, 0.96) | -0.65 (-4.36, 3.12) | **3.08 (0.05, 6.12)** | 0.27 (-1.97, 2.52) | -0.15 (-2.69, 2.4) | -1.59 (-5.3, 2.14) | -1.56 (-3.77, 0.66) | **-2.47 (-4.6, -0.31)** | -0.39 (-4.18, 3.42) | Sildenafil(72.45%) |  |  |
| -0.08 (-3.49, 3.31) | 0.98 (-3.36, 5.3) | -0.06 (-3.78, 3.68) | 1.76 (-2.55, 6.08) | -1.9 (-6.18, 2.38) | -0.82 (-5.18, 3.51) | -0.17 (-3.24, 2.88) | -0.43 (-4.15, 3.26) | 1.03 (-3.27, 5.34) | **4.75 (1.02, 8.47)** | 1.94 (-1.15, 5.03) | 1.52 (-1.81, 4.82) | 0.09 (-4.23, 4.36) | 0.11 (-2.96, 3.17) | -0.8 (-3.82, 2.21) | 1.28 (-3.09, 5.64) | 1.67 (-2.04, 5.36) | Simtuzumab(40.06%) |  |
| -0.98 (-3.32, 1.37) | 0.08 (-3.49, 3.67) | -0.95 (-3.75, 1.87) | 0.86 (-2.69, 4.42) | -2.8 (-6.31, 0.71) | -1.72 (-5.33, 1.87) | -1.07 (-2.9, 0.76) | -1.33 (-4.13, 1.47) | 0.13 (-3.4, 3.68) | **3.85 (1.07, 6.63)** | 1.05 (-0.83, 2.93) | 0.62 (-1.6, 2.86) | -0.82 (-4.32, 2.72) | -0.78 (-2.64, 1.07) | -1.69 (-3.45, 0.07) | 0.38 (-3.21, 4) | 0.77 (-2.01, 3.56) | -0.89 (-4.38, 2.61) | Thalidomide(58.64%) |

### **Supplementary Table S4B league table of VC**

| AZM(34.97%) |  |  |  |  |  |  |  |  |  |  |
| --- | --- | --- | --- | --- | --- | --- | --- | --- | --- | --- |
| 0.32 (-2.6, 3.23) | BUD(29.41%) |  |  |  |  |  |  |  |  |  |
| -0.46 (-2.78, 1.86) | -0.78 (-3.87, 2.33) | Captopril(48.70%) |  |  |  |  |  |  |  |  |
| -0.65 (-3.57, 2.26) | -0.97 (-4.54, 2.6) | -0.19 (-3.3, 2.91) | CTX(53.09%) |  |  |  |  |  |  |  |
| 0.25 (-2.65, 3.13) | -0.07 (-3.61, 3.47) | 0.71 (-2.37, 3.81) | 0.9 (-2.65, 4.46) | IFN(31.10%) |  |  |  |  |  |  |
| -0.78 (-2.34, 0.76) | -1.1 (-3.68, 1.46) | -0.32 (-2.23, 1.57) | -0.13 (-2.72, 2.46) | -1.03 (-3.58, 1.53) | NAC(59.87%) |  |  |  |  |  |
| 0.07 (-2.21, 2.35) | -0.25 (-3.31, 2.82) | 0.54 (-1.99, 3.06) | 0.73 (-2.36, 3.8) | -0.18 (-3.22, 2.87) | 0.86 (-0.97, 2.7) | NAC_MK0476(33.53%) |  |  |  |  |
| -2.52 (-5.49, 0.48) | -2.83 (-6.46, 0.79) | -2.05 (-5.23, 1.12) | -1.86 (-5.48, 1.78) | -2.76 (-6.37, 0.85) | -1.73 (-4.4, 0.94) | -2.58 (-5.75, 0.56) | NAC_RXM(88.8%） |  |  |  |
| -1.23 (-4.1, 1.66) | -1.54 (-5.1, 1.99) | -0.76 (-3.83, 2.33) | -0.57 (-4.11, 2.97) | -1.47 (-4.99, 2.05) | -0.44 (-2.99, 2.12) | -1.3 (-4.33, 1.75) | 1.29 (-2.32, 4.91) | Nintedanib(66.69%) |  |  |
| -1.77 (-3.68, 0.15) | -2.09 (-4.88, 0.73) | -1.3 (-3.5, 0.89) | -1.12 (-3.92, 1.71) | -2.02 (-4.79, 0.78) | -0.98 (-2.34, 0.38) | -1.84 (-3.99, 0.31) | 0.75 (-2.14, 3.64) | -0.54 (-3.31, 2.23) | PFD(83.31%) |  |
| 0.41 (-1.05, 1.85) | 0.08 (-2.43, 2.6) | 0.87 (-0.95, 2.67) | 1.05 (-1.47, 3.59) | 0.16 (-2.34, 2.66) | **1.19 (0.63, 1.76)** | 0.33 (-1.42, 2.08) | **2.92 (0.32, 5.53)** | 1.63 (-0.86, 4.12) | **2.17 (0.93, 3.41)** | Placebo(20.33%) |

### **Supplementary Table S4C league table of TLC**

| Ambroxol(82.52%) |  |  |  |  |  |  |  |  |  |
| --- | --- | --- | --- | --- | --- | --- | --- | --- | --- |
| 1.9 (-0.37, 4.17) | BUD_NAC(22.47%) |  |  |  |  |  |  |  |  |
| 1.21 (-1.22, 3.63) | -0.69 (-2.53, 1.14) | Captopril(48.46%) |  |  |  |  |  |  |  |
| 0.26 (-2.55, 3.07) | -1.64 (-3.97, 0.68) | -0.95 (-3.41, 1.52) | CTX(76.07%) |  |  |  |  |  |  |
| 1.7 (-1.06, 4.47) | -0.21 (-2.47, 2.06) | 0.48 (-1.92, 2.91) | 1.43 (-1.36, 4.23) | IFN(32.68%) |  |  |  |  |  |
| 0.9 (-1.14, 2.89) | -1.01 (-2.28, 0.22) | -0.32 (-1.85, 1.19) | 0.63 (-1.46, 2.69) | -0.8 (-2.83, 1.18) | NAC(62.37%) |  |  |  |  |
| 0.47 (-2.36, 3.27) | -1.44 (-3.75, 0.87) | -0.75 (-3.22, 1.71) | 0.2 (-2.66, 3.04) | -1.23 (-4.03, 1.55) | -0.43 (-2.49, 1.65) | NAC_RXM(70.78%) |  |  |  |
| 1.67 (-1.08, 4.42) | -0.24 (-2.47, 2) | 0.45 (-1.95, 2.86) | 1.4 (-1.39, 4.18) | -0.03 (-2.77, 2.71) | 0.77 (-1.2, 2.77) | 1.2 (-1.6, 3.99) | Nintedanib(33.63%) |  |  |
| 1.09 (-1.18, 3.34) | -0.81 (-2.43, 0.79) | -0.12 (-1.95, 1.71) | 0.83 (-1.48, 3.12) | -0.6 (-2.87, 1.62) | 0.19 (-1.02, 1.44) | 0.63 (-1.67, 2.93) | -0.57 (-2.81, 1.65) | PFD(53.38%) |  |
| 1.95 (-0.01, 3.91) | 0.05 (-1.11, 1.2) | 0.74 (-0.69, 2.17) | 1.68 (-0.33, 3.71) | 0.25 (-1.69, 2.19) | **1.05 (0.58, 1.56)** | 1.48 (-0.51, 3.5) | 0.28 (-1.64, 2.21) | 0.86 (-0.26, 1.99) | Placebo(17.65%) |

### **Supplementary Table S4D league table of DLCO**

| Ambroxol(43.49%) |  |  |  |  |  |  |  |  |  |  |  |  |  |  |  |
| --- | --- | --- | --- | --- | --- | --- | --- | --- | --- | --- | --- | --- | --- | --- | --- |
| -10.85 (-42.49, 20.67) | AZM(74.90%) |  |  |  |  |  |  |  |  |  |  |  |  |  |  |
| -0.06 (-34.59, 34.59) | 10.83 (-13.57, 35.31) | BUD_NAC(42.45%) |  |  |  |  |  |  |  |  |  |  |  |  |  |
| 0.22 (-34.19, 34.93) | 11.11 (-13.38, 35.72) | 0.33 (-27.79, 28.43) | Captopril(41.61%) |  |  |  |  |  |  |  |  |  |  |  |  |
| -3.69 (-43.57, 36.16) | 7.17 (-24.51, 38.99) | -3.64 (-38.24, 30.83) | -3.97 (-38.48, 30.63) | CTX(52.12%) |  |  |  |  |  |  |  |  |  |  |  |
| 0.18 (-39.73, 40.14) | 11.07 (-20.57, 42.88) | 0.27 (-34.34, 34.89) | 0.05 (-34.46, 34.67) | 3.92 (-36.06, 43.78) | IFN(42.94%) |  |  |  |  |  |  |  |  |  |  |
| -5.78 (-34.7, 22.98) | 5.07 (-10.31, 20.31) | -5.76 (-26.41, 14.69) | -6.05 (-26.76, 14.51) | -2.16 (-30.92, 26.57) | -6.01 (-34.84, 22.82) | NAC(63.75%) |  |  |  |  |  |  |  |  |  |
| 0.23 (-31.38, 31.82) | 11.08 (-9, 31.24) | 0.24 (-24.08, 24.64) | -0.06 (-24.41, 24.41) | 3.86 (-27.61, 35.44) | -0.05 (-31.69, 31.65) | 6.03 (-9.11, 21.22) | NAC_MK0476(40.66%) |  |  |  |  |  |  |  |  |
| 0.03 (-39.98, 40.09) | 10.84 (-20.85, 42.51) | 0.02 (-34.54, 34.61) | -0.27 (-35.08, 34.4) | 3.68 (-36.26, 43.55) | -0.29 (-40.19, 39.74) | 5.75 (-22.87, 34.64) | -0.21 (-31.67, 31.36) | NAC_PFD(43.51%) |  |  |  |  |  |  |  |
| -1.13 (-41.27, 38.69) | 9.71 (-21.75, 41.36) | -1.05 (-35.54, 33.39) | -1.42 (-36, 33.12) | 2.47 (-37.37, 42.4) | -1.38 (-41.34, 38.58) | 4.64 (-23.96, 33.39) | -1.34 (-32.83, 30.28) | -1.13 (-40.97, 38.7) | NAC_RXM(46.18%) |  |  |  |  |  |  |
| 0.73 (-31.98, 33.36) | 11.55 (-10.19, 33.49) | 0.74 (-25.04, 26.58) | 0.4 (-25.4, 26.3) | 4.38 (-28.33, 37) | 0.45 (-32.21, 33.29) | 6.49 (-10.75, 23.92) | 0.43 (-21.12, 22.2) | 0.73 (-32.09, 33.44) | 1.81 (-30.65, 34.4) | Nintedanib(39.66%) |  |  |  |  |  |
| -5.71 (-45.86, 34.34) | 5.13 (-26.52, 36.89) | -5.74 (-40.3, 28.86) | -5.95 (-40.95, 28.6) | -2.02 (-42.01, 37.84) | -5.94 (-46.24, 34.12) | 0.05 (-28.74, 29.04) | -5.94 (-37.65, 25.74) | -5.74 (-45.54, 34.3) | -4.57 (-44.58, 35.42) | -6.44 (-39.16, 26.45) | PDN(56.89%) |  |  |  |  |
| 0.37 (-28.86, 29.51) | 11.24 (-4.79, 27.2) | 0.41 (-20.67, 21.47) | 0.11 (-21.07, 21.32) | 4.01 (-25.13, 33.18) | 0.12 (-29.03, 29.38) | 6.16 (-2.73, 15.18) | 0.13 (-15.65, 16.04) | 0.38 (-28.84, 29.51) | 1.52 (-27.5, 30.58) | -0.33 (-17.34, 16.61) | 6.08 (-23.09, 35.43) | PFD(38.84%) |  |  |  |
| 0.73 (-27.55, 29.02) | 11.59 (-2.77, 25.96) | 0.76 (-19.06, 20.64) | 0.46 (-19.48, 20.44) | 4.37 (-23.75, 32.72) | 0.49 (-27.77, 28.83) | **6.53 (1.2, 11.96)** | 0.48 (-13.54, 14.73) | 0.74 (-27.51, 28.94) | 1.88 (-26.25, 29.96) | 0.04 (-16.46, 16.52) | 6.47 (-21.82, 34.84) | 0.37 (-6.78, 7.51) | Placebo(36.53%) |  |  |
| -1.14 (-35.74, 33.52) | 9.73 (-14.86, 34.34) | -1.09 (-29.21, 27.1) | -1.36 (-29.63, 26.85) | 2.52 (-32.15, 37.12) | -1.34 (-35.95, 33.3) | 4.67 (-15.95, 25.41) | -1.36 (-25.7, 23.11) | -1.13 (-35.79, 33.55) | 0 (-34.52, 34.65) | -1.78 (-27.67, 24) | 4.58 (-29.96, 39.34) | -1.48 (-22.72, 19.67) | -1.87 (-21.75, 18.11) | Sildenafil(45.56%) |  |
| -21.67 (-56.86, 13.01) | -10.82 (-35.8, 14.05) | -21.66 (-50.21, 6.7) | -21.96 (-50.59, 6.63) | -18.03 (-53.05, 16.73) | -21.93 (-56.92, 12.86) | -15.9 (-37, 5.27) | -21.91 (-46.75, 2.74) | -21.68 (-56.68, 13.13) | -20.52 (-55.51, 14.18) | -22.41 (-48.75, 3.72) | -16 (-50.96, 18.99) | **-22.09 (-43.78, -0.6)** | **-22.43 (-42.9, -2.07)** | -20.57 (-49.28, 7.86) | Thalidomide(90.93%) |

### **Supplementary Table S4E** **league table of FEV1/FVC**

| AZM(34.14%) |  |  |  |  |  |  |  |  |
| --- | --- | --- | --- | --- | --- | --- | --- | --- |
| 0.18 (-2.81, 3.17) | BUD_NAC(28.46%)) |  |  |  |  |  |  |  |
| -0.97 (-3.37, 1.43) | -1.14 (-3.14, 0.85) | NAC(63.21%) |  |  |  |  |  |  |
| **-4.64 (-8.82, -0.45)** | **-4.82 (-8.77, -0.87)** | **-3.68 (-7.2, -0.15)** | NAC_RXM(97.45%) |  |  |  |  |  |
| -0.47 (-3.73, 2.77) | -0.65 (-3.64, 2.29) | 0.49 (-1.9, 2.86) | 4.16 (0, 8.32) | Nintedanib(46.26%) |  |  |  |  |
| -1.4 (-5.36, 2.6) | -1.57 (-5.31, 2.18) | -0.42 (-3.72, 2.88) | 3.25 (-1.49, 7.99) | -0.91 (-4.87, 3.07) | PDN(64.18%) |  |  |  |
| -0.8 (-3.64, 2.03) | -0.98 (-3.46, 1.51) | 0.16 (-1.58, 1.9) | **3.84 (0.01, 7.66)** | -0.33 (-3.14, 2.48) | 0.59 (-3.02, 4.2) | PFD(56.22%) |  |  |
| 0.3 (-2.01, 2.63) | 0.13 (-1.76, 2.02) | **1.27 (0.64, 1.91)** | **4.95 (1.49, 8.43)** | 0.78 (-1.5, 3.08) | 1.7 (-1.55, 4.93) | 1.11 (-0.52, 2.73) | Placebo(20.51%) |  |
| -0.22 (-3.49, 3.03) | -0.4 (-3.38, 2.58) | 0.75 (-1.64, 3.12) | **4.41 (0.25, 8.57)** | 0.26 (-2.98, 3.51) | 1.17 (-2.82, 5.13) | 0.59 (-2.23, 3.39) | -0.52 (-2.82, 1.76) | Thalidomide(39.58%) |

### **Supplementary Table S5** **A detailed table of adverse drug reaction (ADR)**

| First Author | Year | Country | Treatment(I/C) | | Symptoms and Number of adverse events(I/C) | |
| --- | --- | --- | --- | --- | --- | --- |
|  |  |  |  |  |  |  |
| Zhanmin Zhang | 2005 | China | AZM | Placebo | 3 cases presented with upper abdominal discomfort and loose stools | 3 cases of obesity, 1 case of secondary hypertension, 2 cases of secondary fungal infection |
| Maurits Demedts | 2005 | Belgium | NAC | Placebo | 20 cases of respiratory tract infection, 16 cases of dyspnea, 15 cases of fever, 14 cases of liver-function test abnormal, 13 cases of cough, 12 cases of abdominal pain, 11 cases of upper respiratory tract infection, 9 cases of blood glucose increasd, 6 cases of c-reactive protein increased, 6 cases of blood alkaline phosphatase increased, 6 cases of blood lactate dehydrogenase increased, 6 cases of back pain, 5 cases of respiratory failure, 3 cases of bone marrow toxic effects, 3 cases of edema, 3 cases of headache, 3 cases of asthenia, 3 cases of influenza-like illness, 1 case of muscle cramp | 24 cases of respiratory tract infection, 19 cases of dyspnea, 10 cases of fever, 11 cases of liver-function test abnormal, 16 cases of cough, 7 cases of abdominal pain, 13 cases of upper respiratory tract infection, 11 cases of blood glucose increasd, 3 cases of c-reactive protein increased, 1 case of blood alkaline phosphatase increased, 2 cases of blood lactate dehydrogenase increased, 5 cases of back pain, 1 case of respiratory failure, 10 cases of bone marrow toxic effects, 5 cases of edema, 6 cases of headache, 5 cases of asthenia, 5 cases of influenza-like illness, 4 cases of muscle cramp |
| Shaoxia Liu | 2006 | China | AZM | Placebo | 2 cases experienced nausea and vomiting; 1 case developed a transient rash | / |
| Dafang Wang | 2006 | China | BUD | Placebo | 0 | 5 cases of diabetes, 3 cases of hypertension, 3 cases of electrolyte imbalance |
| Zhigang Yang | 2008 | China | NAC | Placebo | 0 | 2 cases of diabetes, 1 case of osteoporosis |
| Jianyong Zhu | 2009 | China | NAC | Placebo | 2 cases of hypokalemia | 3 cases of hypokalemia |
| Jianyong Zhu | 2009 | China | Captopril | Placebo | 0 | 3 cases of hypokalemia |
| H. Taniguchi | 2010 | Japan | PFD | Placebo | 56 cases of photosensitivity, 18 cases of anorexia, 3 cases of abdominal discomfort, 8 cases of dizziness, 54 cases of nasopharyngitis, 1 case of upper respiratory tract infection, 25 cases of γ-GTP elevation, 4 cases of WBC decrease | 24 cases of photosensitivity, 3 cases of anorexia, 1 case of dizziness, 70 cases of nasopharyngitis, 9 cases of upper respiratory tract infection, 10 cases of γ-GTP elevation |
| H. Taniguchi | 2010 | Japan | PFD | Placebo | 29 cases of photosensitivity, 3cases of eczema asteatotic, 6 cases of anorexia, 4 cases of abdominal discomfort, 30 cases of nasopharyngitis, 3 cases of upper respiratory tract infection, 12 cases of γ-GTP elevation, 3 cases of WBC decrease | 24 cases of photosensitivity, 3 cases of anorexia, 1 case of dizziness, 70 cases of nasopharyngitis, 9 cases of upper respiratory tract infection, 10 cases of γ-GTP elevation |
| Luca Richeldi | 2011 | Italy | Nintedanib | Placebo | 9 cases of diarrhea, 11 cases of cough, 9 cases of nausea, 11 cases of bronchitis, 7 cases of dyspnea, 11 cases of progression of idiopathic pulmonary fibrosis, 1 case of vomiting, 6 cases of upper abdominal pain | 13 cases of diarrhea, 17 cases of cough, 8 cases of nausea, 11 cases of bronchitis, 11 cases of dyspnea, 11 cases of progression of idiopathic pulmonary fibrosis, 4 cases of vomiting, 3 cases of upper abdominal pain |
| Luca Richeldi | 2011 | Italy | Nintedanib | Placebo | 17 cases of diarrhea, 17 cases of cough, 8 cases of nausea, 16 cases of bronchitis, 14 cases of dyspnea, 7 cases of progression of idiopathic pulmonary fibrosis, 6 cases of vomiting, 10s cases of upper abdominal pain | 13 cases of diarrhea, 17 cases of cough, 8 cases of nausea, 11 cases of bronchitis, 11 cases of dyspnea, 11 cases of progression of idiopathic pulmonary fibrosis, 4 cases of vomiting, 3 cases of upper abdominal pain |
| Luca Richeldi | 2011 | Italy | Nintedanib | Placebo | 32 cases of diarrhea, 20 cases of cough, 17 cases of nausea, 7 cases of bronchitis, 13 cases of dyspnea, 9 cases of progression of idiopathic pulmonary fibrosis, 11 cases of vomiting, 2 cases of upper abdominal pain | 13 cases of diarrhea, 17 cases of cough, 8 cases of nausea, 11 cases of bronchitis, 11 cases of dyspnea, 11 cases of progression of idiopathic pulmonary fibrosis, 4 cases of vomiting, 3 cases of upper abdominal pain |
| Luca Richeldi | 2011 | Italy | Nintedanib | Placebo | 47 cases of diarrhea, 8 cases of cough, 20 cases of nausea, 9 cases of bronchitis, 6 cases of dyspnea, 4 cases of progression of idiopathic pulmonary fibrosis, 11 cases of vomiting, 10 cases of upper abdominal pain | 13 cases of diarrhea, 17 cases of cough, 8 cases of nausea, 11 cases of bronchitis, 11 cases of dyspnea, 11 cases of progression of idiopathic pulmonary fibrosis, 4 cases of vomiting, 3 cases of upper abdominal pain |
| Xuemei Yang | 2012 | China | MP | Placebo | 0 | 1 case of mildly elevated blood pressure |
| Sakae Homma | 2012 | Japan | NAC | Placebo | 2 cases of bacterail pneumonia, 1 case of cough, 2 cases of sore throat, 2 cases of hypercholesteraemia | 0 |
| Jianhua Lu | 2013 | China | NAC | Placebo | 0 | 1 case of elevated blood sugar, 1 case of insomnia |
| Haiying Zhang | 2014 | China | PDN | Placebo | 11 cases of hypertension, 6 cases of gastrointestinal bleeding, 1 case of bone disease, 1 case of muscle atrophy, 1 case of depression | 11 cases of hypertension, 7 cases of gastrointestinal bleeding, 4 cases of bone disease, 4 cases of muscle atrophy, 4 cases of depression |
| Fernando J Martinez | 2014 | USA | NAC | Placebo | 9 cases of respiratory, 6 cases of infectious, 9 cases of cardiac, | 9 cases of respiratory, 6 cases of infectious, 2 cases of cardiac, 6 cases of gastrointestinal |
| Luca Richeldi | 2014 | UK | Nintedanib | Placebo | 190 cases of diarrhea, 70 cases of nausea, 39 cases of nasopharyngitis, 47 cases of cough, 31 cases of progression of idiopathic pulmonary fibrosis, 36 cases of bronchitis, 28 cases of upper respiratory tract infection, 22 cases of dyspnea, 26 cases of decreased appetite, 40 cases of vomiting, 25 cases of weight loss | 38 cases of diarrhea, 12 cases of nausea, 34 cases of nasopharyngitis, 26 cases of cough, 21 cases of progression of idiopathic pulmonary fibrosis, 28 cases of bronchitis, 18 cases of upper respiratory tract infection, 23 cases of dyspnea, 14 cases of decreased appetite, 4 cases of vomiting, 13 cases of weight loss |
| Luca Richeldi | 2014 | UK | Nintedanib | Placebo | 208 cases of diarrhea, 86 cases of nausea, 48 cases of nasopharyngitis, 38 cases of cough, 33 cases of progression of idiopathic pulmonary fibrosis, 31 cases of bronchitis, 30 cases of upper respiratory tract infection, 27 cases of dyspnea, 42 cases of decreased appetite, 34 cases of vomiting, 37 cases of weight loss | 40 cases of diarrhea, 16 cases of nausea, 34 cases of nasopharyngitis, 31 cases of cough, 40 cases of progression of idiopathic pulmonary fibrosis, 17 cases of bronchitis, 24 cases of upper respiratory tract infection, 25 cases of dyspnea, 10 cases of decreased appetite, 7 cases of vomiting, 2 cases of weight loss |
| Qingyan Huang | 2015 | China | NAC | Placebo | 2 cases of eyelid swelling, 1 case of wheezing | 1 case of osteoporosis, 1 case of chest tightness |
| Shanguo Jiang | 2015 | China | BUD+NAC | Placebo | 0 | 0 |
| Wei Jin | 2015 | China | NAC | Placebo | 0 | 0 |
| Huiping Li | 2015 | China | PFD | Placebo | 36 adverse reactions, details unspecified | 32 adverse reactions, details unspecified |
| Yong Liu | 2015 | China | NAC | Placebo | 3 cases of edema, 2 cases of gastrointestinal discomfort, 1 case of elevated blood pressure, 1 case of oral infection | 2 cases of edema, 1 case of elevated blood pressure, 1 case of elevated blood glucose |
| Yan Shen | 2015 | China | AZM | Placebo | 3 cases experienced mild discomfort symptoms such as nausea, vomiting, and throat itching | 2 cases experienced mild discomfort symptoms such as nausea, vomiting, and throat itching |
| Chuanhai Wang | 2015 | China | NAC | Placebo | 3 cases experienced symptoms of nausea and vomiting | 0 |
| Li Zhao | 2015 | China | NAC | Placebo | 2 cases of elevated blood glucose, 2 cases of hypertension, 3 cases of gastrointestinal discomfort | 4 cases of elevated blood glucose, 3 cases of elevated blood pressure, 1 case of gastrointestinal bleeding, 3 cases of electrolyte imbalance, and 2 cases of osteoporosis |
| Hui Huang | 2015 | China | PFD | Placebo | 16 cases of skin-related, 24 cases of gastrointestinal-related, 5 cases of laboratory abnormalities | 6 cases of skin-related, 8 cases of gastrointestinal-related, 0 case of laboratory abnormalities |
| Yunxia Li | 2016 | China | PFD | Placebo | 1 case of severe gastrointestinal reaction | 0 |
| Zheng Lu | 2016 | China | SASH | Placebo | 0 | 0 |
| Weihua Tian | 2016 | China | NAC | Placebo | 2 cases of concurrent infection 1 case of nausea and vomiting | 3 cases of concurrent infection, 1 case of gastrointestinal ulcer |
| Chuanhai Wang | 2016 | China | NAC+MK0476 | Placebo | 3 cases of nausea and vomiting, 1 case of dizziness | / |
| Feng Xu | 2016 | China | NAC+MK0476 | Placebo | 2 cases of dizziness and nausea | 0 |
| Jürgen Behr | 2016 | Germany | NAC | Placebo | 1 case of back pain, 2 cases of intervertebral discprotrusion, 1 case of diarrhoea, 1 case of sinusitis, 1 case of headache, 1 case of hypertension, 1 case of dyspnoea, 1 case of headache, 1case of hypertension, 1 case of malignant lung neoplasm | 2 cases of forearm fracture, 1 case of aortic aneuysm, 1 case of aortic aneurysm, 1 case of contusion, 1 case of worsening IPF |
| Hongmei Chen | 2017 | China | NAC | Placebo | 1 case of choking cough, 1 case of nausea | 1 case of choking cough, 2 cases of nausea, 2 cases of vomiting, 2 cases of diarrhea |
| Qingshuang Mu | 2017 | China | NAC | Placebo | 0 | 4 cases of mild gastrointestinal reactions, 2 cases of elevated blood glucose |
| Arata Azuma | 2017 | Japan | Nintedanib | Placebo | 57 cases of diarrhoea, 27 cases of nasopharyngitis, 10 cases of origression of IPF, 15 cases of nausea, 14 cases of decreased appetite, 14 cases of abnormal hepatic function, 12 cases of increased hepatic enzyme, 11 cases of bronchitis, 11 cases of decreased weight, 2 cases of diabetes mellitus, 10 cases of constipation, 10 cases of vomiting, 3 cases of abdominal discomfort, 3 cases of back pain | 9 cases of diarrhoea, 19 cases of nasopharyngitis, 13 cases of origression of IPF, 4 cases of decreased appetite, 1 case of abnormal hepatic function, 2 cases of increased hepatic enzyme, 7 cases of bronchitis, 3 cases of decreased weight, 7 cases of diabetes mellitus, 2 cases of constipation, 6 cases of abdominal discomfort, 5 cases of back pain |
| Arata Azuma | 2017 | Japan | Nintedanib | Placebo | 398 cases of diarrhoea, 87 cases of nasopharyngitis, 64 cases of origression of IPF, 156 cases of nausea, 68 cases of decreased appetite, 17 cases of abnormal hepatic function, 21 cases of increased hepatic enzyme, 67 cases of bronchitis, 62 cases of decreased weight, 4 cases of diabetes mellitus, 38 cases of constipation, 74 cases of vomiting, 15 cases of abdominal discomfort, 37 cases of back pain | 78 cases of diarrhoea, 68 cases of nasopharyngitis, 61 cases of origression of IPF, 28 cases of nausea, 24 cases of decreased appetite, 1 case of abnormal hepatic function, 2 cases of increased hepatic enzyme, 45 cases of bronchitis, 15 cases of decreased weight, 12 cases of diabetes mellitus, 17 cases of constipation, 11 cases of vomiting, 8 cases of abdominal discomfort, 29 cases of back pain |
| Ganesh Raghu | 2017 | USA | Simtuzumab | Placebo | 259 adverse reactions, details unspecified | 263 adverse reactions, details unspecified |
| Kaichun Lei | 2018 | China | PFD | Placebo | 13 cases exhibited adverse reactions, including mild to moderate photosensitivity reactions, decreased appetite, abdominal discomfort, and fatigue | 0 |
| Jingpan Li | 2018 | China | NAC | Placebo | 0 | 1 patient experienced vomiting. |
| Zhenxing Mao | 2018 | China | PFD | Placebo | 3 cases of nausea, 4 cases of vomiting, 4 cases of photosensitivity, 1 case of hemoptysis | 2 cases of nausea, 3 cases of vomiting, 2 cases of gastritis |
| Martin Kolb | 2018 | Canada | Sildenafil | Placebo | 79 cases of diarrhea, 22 cases of nausea, 21 cases of headache, 20 cases of decreased appetite, 20 cases of cough, 19 cases of vomiting, 18 cases of dyspnea | 66 cases of diarrhea, 14 cases of nausea, 10 cases of headache, 23 cases of decreased appetite, 13 cases of cough, 10 cases of vomiting, 13 cases of dyspnea |
| Carlo Vancheri | 2018 | Italy | PFD | Placebo | 20 cases of diarrhea, 22 cases of nausea | 16 cases of diarrhea, 6 cases of nausea |
| Lu Cao | 2018 | China | NAC | Placebo | 2 cases of pulmonary infection, 2 cases of dizziness, 1 case of gastric ulcer | 2 cases of dizziness, 2 cases of gastric ulcer |
| Shimou Chen | 2019 | China | NAC | Placebo | 2 cases of concurrent infection, 1 case of nausea and vomiting | 4 cases of concurrent infections, 2 cases of gastrointestinal ulcers |
| Junxing Zhu | 2019 | China | BUD | Placebo | 1 case of hypertension, 1 case of stomatitis | 2 cases of hypertension, 1 case of fungal infection, 3 cases of stomatitis, 1 case of obesity |
| Chunlian Liang | 2019 | China | PFD | Placebo | 6 cases of gastrointestinal symptoms, 2 cases of rash and photosensitivity, 3 cases of elevated transaminases | 0 |
| Fenli Wang | 2019 | China | PFD | Placebo | 2 cases of nausea, 1 case of vomiting, 1 case of anorexia, 1 case of rash | 1 case of nausea, 1 case of anorexia |
| Jianying Wen | 2019 | China | NAC | Placebo | 0 | 0 |
| Chengjie Zhao | 2019 | China | NAC+PFD | Placebo | 0 | 0 |
| Zhenzhen Zhang | 2020 | China | PFD | Placebo | 3 adverse reactions, details unknown | 2 adverse reactions, details unknown |
| Lisa Lancaster | 2020 | USA | Nintedanib | Placebo | 38 cases of diarrhea, 16 cases of nausea, 10 cases of fatigue, 7 cases of headache, 9 cases of decreased appetite, 4 cases of cough, 9 cases of vomiting | 18 cases of diarrhea, 11 cases of nausea, 6 cases of fatigue, 6 cases of headache, 4 cases of decreased appetite, 7 cases of cough, 2 cases of vomiting |
| Luca Richeldi | 2020 | Italy | Nintedanib | Placebo | 335 cases of diarrhea, 145 cases of nausea, 53 cases of decreased appetite, 62 cases of nasopharyngitis, 61 cases of cough, 61 cases of vomiting, 30 cases of dyspnoea, 33 cases of progression of IPF, 41 cases of weight decreased, 53 cases of abominal pain, 90 cases of adverse events leading to treatment | 68 cases of diarrhea, 25 cases of nausea, 16 cases of decreased appetite, 43 cases of nasopharyngitis, 35 cases of cough, 11 cases of vomiting, 25 cases of dyspnoea, 34 cases of progression of IPF, 8 cases of weight decreased, 6 cases of abominal pain, 32 cases of adverse events leading to treatment |
| Luca Richeldi | 2020 | Italy | Pamrevlumab | Placebo | 48 adverse reactions, primarily related to gastrointestinal reactions | 52 adverse reactions, primarily related to gastrointestinal reactions |
| Jin Woo Song | 2020 | South Korea | Nintedanib | Placebo | 153 cases of diarrhoea, 50 cases of nasopharyngitis, 42 cases of nausea, 25 cases of progression of IPF, 34 cases of upper respiratory tract infection, 10 cases of lung infection, 30 cases of decreased appetite, 23 cases of bronchitis, 19 cases of cough | 27 cases of diarrhoea, 37 cases of nasopharyngitis, 2 cases of nausea, 27 cases of progression of IPF, 24 cases of upper respiratory tract infection, 6 cases of lung infection, 12 cases of decreased appetite, 8 cases of bronchitis, 11 cases of cough |
| Xiaodan Zhou | 2021 | China | NAC | Placebo | 4 cases of elevated, 5 cases of gastrointestinal symptoms, 1 case of light allergic | 4 cases of elevated, 6 cases of gastrointestinal symptoms, 1 case of light allergic, 1 case of skin lesions |
| Ze Deng | 2022 | China | Nintedanib | Placebo | 5 cases of nausea and vomiting, 2 cases of fatigue, 1 case of loss of appetite, 1 case of diarrhea | 4 cases of nausea and vomiting, 1 case of fatigue, 2 cases of loss of appetite |
| Zhong Guo | 2022 | China | NAC | Placebo | 1 case of vomiting, 1 case of nausea, 1 case of infection | 2 cases of peptic ulcer disease， 2 cases with superimposed infection |
| Lu Song | 2022 | China | Nintedanib | Placebo | 1 case of dizziness, 1 case of rash | 1 case of nausea |
| Zehai Xia | 2022 | China | PFD | Placebo | 6 cases of upper abdominal discomfort, 1 case of hepatic dysfunction, 3 cases of photosensitive dermatitis, 2 cases of pruritus, 2 cases of fatigue, 1 case of weight loss | / |
| Weiwei Chen | 2023 | China | PFD | Placebo | 2 cases of nausea and vomiting, 1 case of skin injury, 1 case of elevated transaminase levels | 1 case of nausea and vomiting 1 case of skin injury |
| Haitao Hu | 2023 | China | PFD | Placebo | 2 cases of nausea, 2 cases of loss of appetite, 1 case of rash | 3 cases of nausea, 2 cases of loss of appetite, 1 case of rash |
| Shuai Wang | 2023 | China | Nintedanib | Placebo | 3 cases of gastritis, 6 cases of nausea, 6 cases of vomiting, 5 cases of photosensitivity reaction | 2 cases of gastritis, 3 cases of nausea, 4 cases of vomiting, 3 cases of photosensitivity reaction |
| Chanchan Xu | 2023 | China | NAC | Placebo | 0 | 0 |
| Hongmei Zhao | 2023 | China | NAC | Placebo | 3 cases of photosensitivity reaction | 1 cases of photosensitivity reaction |
| Haiyan Chen | 2024 | China | Ambroxol | Placebo | 0 | 0 |
| Zhenxiang Liu | 2024 | China | NAC | Placebo | 1 case of nausea, 1 case of vomiting | 4 cases of nausea, 4 cases of vomiting, 2 cases of hemoptysis |
| Huanqin Wang | 2024 | China | PFD | Placebo | 3 cases of gastrointestinal reactions, 1 case of secondary infection, 2 cases of dizziness and headache, 1 case of mental abnormality | 2 cases of gastrointestinal reactions, 1 case of secondary infection, 2 cases of dizziness and headache, 2 cases of mental abnormalities |
| Xizhi Wang | 2024 | China | NAC | Placebo | 1 case of upper respiratory tract infection, 3 cases of loss of appetite, 3 cases of abdominal discomfort, 2 cases of rash | 2 cases of loss of appetite, 2 cases of abdominal discomfort, 2 cases of rash |
| Lifei Yue | 2024 | China | PFD | Placebo | 8 cases of gastrointestinal reactions, details unspecified | 6 cases of gastrointestinal reactions, details unspecified |
| Yanni Zhang | 2024 | China | Metformin | Placebo | 1 case of nausea, 1 case of vomiting, 2 cases of upper abdominal discomfort | 1 case of nausea, 1 case of vomiting, 1 case of upper abdominal discomfort |
| Jing Zhao | 2024 | China | Thalidomide | Placebo | 3 cases of nausea and vomiting, 1 case of diarrhea, 1 case of rash | 4 cases of nausea and vomiting, 2 cases of diarrhea, 1 case of rash, 1 case of dizziness |
| Ganesh Raghu | 2024 | USA | Pamrevlumab | Placebo | 23 cases of serious adverse reactions, mostly related to gastrointestinal reactions, with specific details unavailable. | 22 cases of serious adverse reactions, mostly related to gastrointestinal reactions, with specific details unavailable. |
